# A Flp-SUMO hybrid recombinase reveals multi-layered copy number control of a selfish DNA element through post-translational modification

**DOI:** 10.1371/journal.pgen.1008193

**Published:** 2019-06-26

**Authors:** Chien-Hui Ma, Bo-Yu Su, Anna Maciaszek, Hsiu-Fang Fan, Piotr Guga, Makkuni Jayaram

**Affiliations:** 1 Department of Molecular Biosciences, University of Texas at Austin, Austin, TX, United States of America; 2 Department of Life Sciences and Institute of Genome Sciences, Biophotonics and Molecular Imaging Research Center, National Yang-Ming University, Taipei City, Taiwan; 3 Centre of Molecular and Macromolecular Studies, Polish Academy of Sciences, Department of Bioorganic Chemistry, Lodz, Poland; Fred Hutchinson Cancer Research Center, UNITED STATES

## Abstract

Mechanisms for highly efficient chromosome-associated equal segregation, and for maintenance of steady state copy number, are at the heart of the evolutionary success of the 2-micron plasmid as a stable multi-copy extra-chromosomal selfish DNA element present in the yeast nucleus. The Flp site-specific recombination system housed by the plasmid, which is central to plasmid copy number maintenance, is regulated at multiple levels. Transcription of the *FLP* gene is fine-tuned by the repressor function of the plasmid-coded partitioning proteins Rep1 and Rep2 and their antagonist Raf1, which is also plasmid-coded. In addition, the Flp protein is regulated by the host’s post-translational modification machinery. Utilizing a Flp-SUMO fusion protein, which functionally mimics naturally sumoylated Flp, we demonstrate that the modification signals ubiquitination of Flp, followed by its proteasome-mediated degradation. Furthermore, reduced binding affinity and cooperativity of the modified Flp decrease its association with the plasmid *FRT* (Flp recombination target) sites, and/or increase its dissociation from them. The resulting attenuation of strand cleavage and recombination events safeguards against runaway increase in plasmid copy number, which is deleterious to the host—and indirectly—to the plasmid. These results have broader relevance to potential mechanisms by which selfish genomes minimize fitness conflicts with host genomes by holding in check the extra genetic load they pose.

## Author summary

Plasmids of budding yeasts, exemplified by the 2-micron plasmid of *Saccharomyces cerevisiae*, and mammalian papilloma and gammaherpes viruses typify eukaryotic extra-chromosomal selfish DNA elements. The plasmid and the viral episomes, despite the long evolutionary divergence of their hosts, share striking similarities in lifestyles. These include the ability to segregate to daughter cells by hitchhiking on chromosomes and to switch from cell cycle regulated replication to iterative replication for copy number maintenance. While selfish elements, including those integrated into chromosomes, rely on their hosts’ genetic potential for long-term survival, their genetic load is carefully regulated to minimize fitness conflicts with the hosts. Our study focuses on the Flp site-specific recombinase, which is central to the copy number control of the 2-micron plasmid and whose steady state levels are regulated through transcriptional control by plasmid coded proteins and through post-translational modification by the host’s sumoylation machinery. We demonstrate that sumoylation, in addition, attenuates the catalytic activity of Flp by diminishing its DNA binding affinity and inter-monomer cooperativity, providing another layer of protection against runaway increase in plasmid copy number. Population control by self-imposed and host-mediated mechanisms is likely a general strategy among selfish elements to ensure nearly conflict-free coexistence with host genomes.

## Introduction

The yeast 2-micron plasmid, nearly ubiquitous among *Saccharomyces* yeast strains, is a highly optimized extrachromosomal selfish DNA element [1–4]. The plasmid resides in the nucleus, offers no apparent fitness advantage to its host, and does not impose any significant disadvantage at its normal copy number of 40–60 molecules per haploid chromosome set. The compact plasmid genome (~6.3 kbp) is organized into two functional modules, one devoted to stable propagation (the plasmid partitioning system) and the other to copy number maintenance (the plasmid amplification system).

The partitioning system [5–7], comprised of the plasmid-coded Rep1 and Rep2 proteins together with a *cis*-acting locus *STB*, promotes nearly equal segregation of plasmid molecules duplicated by the host replication machinery into mother and daughter cells. Current evidence is consistent with a ‘hitchhiking model’ in which the plasmid utilizes chromosomes as a vehicle for segregation by physically associating with them [8–11]. In this respect, the plasmid resembles the episomes of mammalian papilloma and gammaherpes viruses that also resort to chromosome-tethering for stable maintenance during prolonged periods of latent infection [12–20]. It is possible that selfish genomes inhabiting evolutionarily distant hosts have independently converged on the common strategy of chromosome-coupled segregation as a means for self-preservation.

**Fig 1:**
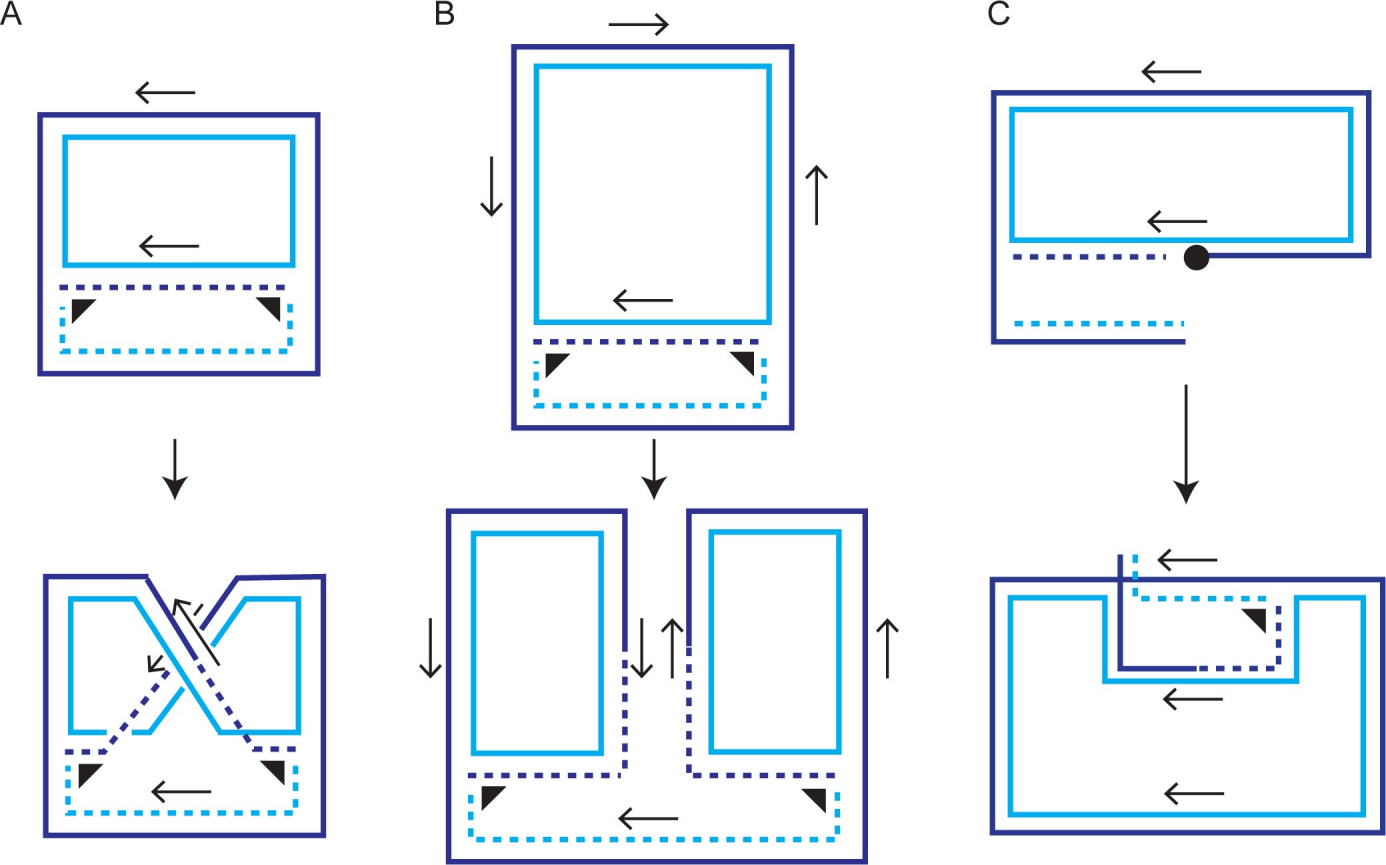
Amplification of the 2-micron plasmid by the action of the plasmid coded Flp site-specific recombinase may occur by more than one mechanism. Three possible modes of plasmid amplification are illustrated in **A**-**C**. In **A**, site-specific recombination between the head-to-head Flp target sites (*FRT*s; thin arrows) of a replicating plasmid monomer sets up a pair of replication forks (thick arrowheads) that chase each other along the circular template [21]. In **B**, site-specific recombination between two head-to-tail target sites in a replicating plasmid dimer generates two interconnected uni-directionally replicating circular monomers [23]. In **C**, the double strand break arising from a Flp-induced single strand nick within an *FRT* site of a replicating plasmid triggers break induced replication (BIR) by strand invasion of a second plasmid circle [31,33]. The circular knob indicates Flp covalently linked to the 3’-phosphate terminus of the cleaved strand. The unifying feature in **A**-**C** is the establishment of non-terminating uni-directional replication of circular DNA molecules as a result of a successful (**A**, **B**) or an aborted (**C**) site-specific recombination event. Plasmid amplification by the schemes in **A** and **B** may be regulated by a second recombination event to restore replication termination. Amplification by the scheme in **C** is more difficult to regulate, and likely involves the resolution of branched DNA intermediates formed by the encounter between two BIR forks or between a BIR fork and a replication fork [53] (see also the schematic diagram in Fig 3A).

The plasmid amplification system, consisting of the plasmid-coded Flp site-specific recombinase and its target *FRT* sites arranged in head-to-head orientation within the plasmid genome, counteracts any reduction in copy number resulting from rare missegregation events [21,22]. Amplification is thought to be triggered by a Flp-mediated recombination event coordinated with bi-directional plasmid replication—DNA inversion within a plasmid monomer or resolution within a plasmid dimer—that reconfigures the mode of replication (Fig 1A and 1B) [21,23]. A second recombination event can restore normal fork movement, and terminate amplification. The amplified plasmid concatemer may be resolved into monomers by Flp or by the host’s homologous recombination machinery. Positive and negative transcriptional regulation of *FLP* by plasmid-coded proteins—the putative Rep1-Rep2 repressor and its antagonist Raf1—ensures a prompt amplification response when needed without causing a runaway increase in plasmid copy number [24–27]. Thus, self-imposed moderation of selfishness is an integral element in the survival strategy of the yeast plasmid [2,28,29]. Interestingly, Raf1 appears to play a dual role in plasmid physiology, contributing to both plasmid stability and copy number control. In addition to blocking the assembly of the Rep1-Rep2 repressor complex, Raf1 is involved in promoting the organization of the Rep1-Rep2-*STB* partitioning complex [26,30].

The post-translational protein modification machinery of the host also contributes to the regulation of 2-micron plasmid stability and copy number [31–33]. Impaired sumoylation of Rep1 and Rep2 interferes with their *STB*-association, and adversely affects plasmid segregation [32]. Deficient SUMO conjugation to Flp raises its steady-state levels, leading to hyper-amplification of the plasmid. The resulting increase in plasmid load causes cell cycle delays and reduced replicative life-span [31,34,35]. The 2-micron plasmid exemplifies the collaborative roles of self-regulation and host-mediated regulation in the coexistence of a selfish DNA element and its host genome with minimal mutual conflicts between them.

High plasmid copy number and attendant cell death phenotypes are produced by a variety of mutations in protein components associated with SUMO conjugation and deconjugation steps, and with ubiquitin-dependent degradation of sumoylated proteins. These mutations map to E3 ligases (*siz1Δ*, *siz2Δ*), the SUMO maturase/deconjugase (*ulp1* or *nib1*), a SUMO-targeted ubiquitin ligase (*slx5Δ*, *slx8Δ*) and certain NPC (nuclear pore complex) proteins required for normal cellular localization of Ulp1 [31,33,34,36–38]. These mutants exhibit a marked differential killing effect on yeast strains harboring the 2-micron plasmid [Cir^+^] versus those lacking the plasmid [Cir^0^]. The misregulated amplification of plasmid DNA likely stems from enhanced single strand nicks at the plasmid *FRT* sites due to elevated Flp levels (Fig 1C) [31,33]. Conversion of the nick into a double strand break by encountering an advancing replication fork can trigger strand invasion by the broken end into an intact circular plasmid, to be followed by break-induced replication (BIR) (Fig 1C). In principle, BIR in the circular template may persist through multiple rounds, producing large plasmid concatemers. BIR-mediated aberrant amplification is supported by the significant reduction in a high molecular weight DNA form of the plasmid in the absence of Pol32 or of Rad proteins required for known BIR pathways [33]. The reaction is formally analogous to the alternative (telomerase-independent) pathway for lengthening of telomeres *via* telomere mini-circles as templates, which occurs in yeast, many transformed cell lines and in certain human cancers [39,40].

We wished to address whether, in addition to lowering Flp levels, the SUMO modification of Flp may also modulate its DNA recognition and/or catalytic properties. To circumvent the technical challenges posed by the low level of the *in vivo* modification, we utilized a Flp-SUMO fusion protein in which SUMO residues 1–96 are joined in frame to the carboxyl-terminus of Flp. By demonstrating the nearly identical behavior Flp-SUMO and physiologically sumoylated Flp in a variety of *in vivo* experimental contexts, we validated the utility of the fusion protein in directly probing the effects of SUMO modification on the physicochemical interactions of Flp with the *FRT* site. *In vitro* assays using purified Flp and Flp-SUMO revealed that Flp-SUMO binds *FRT* less efficiently than Flp and with weaker cooperativity, and is preferentially excluded from *FRT* in the presence of Flp. Consequently, the fusion protein is less active in *FRT* x *FRT* recombination than Flp, and the lower activity is reflected in both the strand cleavage and strand joining steps of recombination. The *in vitro* results are corroborated by an *in vivo* assay for DNA damage induced by Flp and Flp-SUMO at *FRT* sites, consistent with the lower occupancy of these sites (or accelerated exit from them) by the fusion protein.

The present results, in conjunction with previously published reports [24,25,27,31,33], suggest a tripartite mechanism for the copy number control of the 2-micron plasmid involving gene expression, protein turnover and protein activity. The first is imposed by the plasmid itself, while the other two are instituted by the host. Collectively, they provide a paradigm for the bilateral interactions through which selfish DNA elements and their host organisms strike a fine balance between the fitness advantage gained by such an element from high copy number and the fitness cost incurred by the host—and thus indirectly by the element—from the extra genetic load.

## Materials and methods

### Yeast strains and Plasmids

The list of yeast strains and plasmids utilized in this study is given in S1 and S2 Tables, respectively. The specific figures and table depicting the experiments in which they were employed are also indicated. The presence or absence of the native 2-micron plasmid in a given strain is denoted as [Cir^+^] or [Cir^0^], respectively. This designation does not include *ARS*-based or 2-micron-derived plasmid constructs. The genotype of a strain containing such engineered plasmids, but not the native plasmid, is still referred to as [Cir^0^] with the resident plasmid spelled out.

### Construction of yeast plasmids

The yeast plasmids (S1 Fig) used for genetic assays were constructed by a strategy analogous to that described previously [41]. The rationale is to generate two requisite linear DNA fragments *in vitro* by PCR amplification, and allow them to self-assemble the desired circular plasmid *in vivo* by homologous recombination/repair. Recombination is facilitated by overlapping sequences that these fragments carry at their ends. A suitable marker (*ADE2*) contained in one of the fragments permits the selection of plasmid-containing cells. We used a constant DNA fragment corresponding to the A-form of the plasmid [42] that included, in sequential order, the 2-micron plasmid *RAF1* gene, *ADE2* inserted into the plasmid HpaI site, *STB*, *ORI*, a copy of the inverted repeat, and the *REP2* gene. The other variable fragment included the *REP1* gene, the second copy of the inverted repeat, and the *FLP* gene with the incorporated modifications. Sequences adjoining *REP1* and *RAF1* provided homology at one end. Homology at the other end came from sequences adjacent to *FLP* and *REP2*. An equimolar mixture of the constant fragment with one of the variable fragments was used to transform an *ade2* [Cir^0^] yeast strain to adenine prototrophy. DNA samples isolated from a subset of the transformants were analyzed by PCR to identify those that contained the correct plasmid. Critical regions of the plasmid, including the modified *FLP* locus and the recombination junction regions, were further verified by DNA sequencing. Once a parental strain harboring the correct plasmid was established, subsequent transfer of the plasmid to other recipient strains was performed by transformation using isolated total DNA.

### Engineering yeast chromosomes

Integration of exogenous DNA cassettes into a specific chromosome locale was accomplished by one of three methods based on homology-dependent double strand break repair: (1) using a linearized integrative plasmid cut within the region of homology, (2) using PCR-amplified DNA fragments with flanking homology, or (3) using the CRISPR (Cas9-sgRNA) technology. The first two methods required selection of a marker included in the incoming/editing DNA; no selection was required for the third method. All constructs were authenticated by DNA sequencing.

### Purification of Flp and Flp-SUMO

Native Flp, Flp-HA-His8 and Flp-SUMO-HA-His8, as well as mutant derivatives of the tagged proteins, were overexpressed in *E*. *coli* cells using the pBAD system (Invitrogen). Purification of untagged Flp was carried out using previously described procedures [43–45]. Purification of the tagged proteins included an additional first step of nickel chromatography, followed by dialysis to remove the imidazole present in the elution buffer. The final preparations were ≥ 85% pure, as judged by SDS-PAGE and densitometric scanning of the Coomassie Blue stained bands. Protein concentrations in the final preparations were determined using the Bradford assay.

### Analyses of colony morphology and plasmid loss

Overnight cultures were prepared from purified single transformant colonies containing individual 2-micron circle-derived plasmids by growing them selectively (in medium lacking adenine) at 30°C. These cultures were diluted in YEPD medium (n = 0; 10^4^ cells/ml) and grown for 10 generations (n = 10) at 30°C. Aliquots were plated out from n = 0 and n = 10 cultures on YEPD medium. Nibbled and mini-colonies were counted after incubating the plates for 5 days at 26°C. A founder cell that had lost the plasmid, and the plasmid-borne *ADE2* marker, gave rise to a fully red (non-sectored) smooth colony. Such colonies were excluded from the total population in calculating the fraction of nibbled colonies. Plasmid loss rate per generation ‘I’ (for instability) was estimated from plates incubated at 30°C based on fully red and total colony counts. I = (1/10) x [ln (f_0_/f_10_)] [46], where f_0_ and f_10_ are the fractions of plasmid containing cells (yielding colonies other than the fully red ones) at n = 0 and n = 10, respectively. The sample size for the individual estimates of plasmid loss rate and the fraction of nibbled or mini-colonies was a minimum of 800 colonies. The assays were performed as at least three repetitions.

### Real-time PCR for plasmid copy number determination

The PCR protocols followed those described by Chen et al. [31], and utilized the same plasmid and reference chromosomal amplicons as well as the primer pairs described by them. The amplification reactions were carried out with ABI PRISM 7900HT SDS using the SYBR Green Master Mix (Applied Biosystems). The number of cycles required to reach the *C*_*T*_ number (preset threshold) for each DNA sample was calculated from six separate experiments. The relative change in the copy number of a plasmid between two strains, normalized to the chromosomal reference sequence, was calculated by the 2^-*ΔΔCT*^ analysis [47].

### Protein stability assays

The expression cassette for Flp-SUMO controlled by the *GAL1* promoter was inserted at the *TRP1* locus (thus disrupting it) on chromosome IV in [Cir^0^] wild type, *siz1Δ siz2Δ*, *slx5Δ* and *slx8Δ* strains. Aliquots of raffinose-grown overnight cultures were inoculated into raffinose medium, grown to mid-log phase at 30°C, and induction was performed by transferring them to 2% galactose with continued incubation at 30°C. The control (uninduced) cells were transferred from raffinose to glucose medium and incubated at 30°C. At 2hr, cells were spun down, washed, and suspended in TE buffer before adding cycloheximide (100 μg/ml) to arrest protein synthesis. Cells removed at intervals over a 60 min time course were treated with lysis buffer (50 mM HEPES, pH 7.0; 75 mM KCl., 1 mM MgCl_2_, 1 mM EGTA, 0.5% Triton X-100, I mM DTT, 1 mM PMSF and one protease inhibitor tablet from Roche/50 ml). Cell extracts prepared by bead beating (5 min; 4°C) were fractionated by 12% SDS-PAGE, and analyzed by quantitative western blotting. Flp-SUMO bands were detected by anti-HA antibody (BioLegend) at 1:1000 dilution, and normalized against actin bands visualized using anti-β-actin antibody (Gene Tex) at 1:1000 dilution.

### Protein stability upon proteasome inhibition by MG-132

Proteasome function was inhibited with MG-132 according to published procedures [48]. The following modifications were made to the standard protocols for measuring protein turnover. Overnight raffinose cultures were grown in synthetic medium without ammonium sulfate, and supplemented with 0.1% proline as well as other appropriate amino acids. In addition, the re-inoculation medium for obtaining mid-log phase cells for galactose induction included 0.003% SDS. The induction period was 2 hr, with 75 μm MG-132 (Biomol, Plymouth Meeting, PA) being added at 90 min. Control cells received an equivalent volume of DMSO, the solvent for MG-132. The rest of the procedure—cycloheximide treatment, preparation and fractionation of cell extracts, and western blotting—was performed as described under the previous section on protein stability assays.

### Rad52 foci assay for double strand DNA breaks

The experimental strains were derived from [Cir^0^] wild type and *siz1Δ siz2Δ* strains expressing the *GAL1* promoter driven Flp-SUMO (see the section above on protein stability assays) or from an analogous set of strains in which Flp-SUMO was replaced by Flp. The plasmid p*ADE2*-Flp (S1 Fig) was introduced into these strains, and maintained by adenine selection. A *CEN*-*TRP1*-plasmid expressing Rad52-YFP from the native *RAD52* promoter was also maintained in them by selection. The conditions for Flp or Flp SUMO induction were the same as those described for protein stability estimates in the absence of MG-132. Cells induced for 2 hr in galactose and the corresponding uninduced control cells (2 hr in glucose) were collected, washed and fixed in formaldehyde for scoring fluorescent foci. Each set of assays was repeated three times.

### *In vitro FRT*-binding assays with Flp or Flp-SUMO

The binding assays were performed in 30 μl individual mixtures incubated on ice for 20 min using the buffer conditions described by Prasad et al. [49]. The substrate DNA fragment (0.05 pmol per binding reaction) was 262 bp long, and contained one *FRT* site. Aliquots were fractionated by electrophoresis in 5% polyacrylamide gels (29:1 crosslinking) at 4°C in 1x TBE duffer. The bound complexes and the unbound substrate were visualized by autoradiography or phosphor imaging.

### Recombination, strand cleavage and strand joining assays

The conditions for *in vitro* recombination were similar to those described previously [50,51]. Each 30 μl reaction mixture contained 0.2 pmol plasmid substrate (with two *FRT* sites oriented head-to-tail) and 1 pmol of purified Flp or Flp-SUMO. At the end of the 30°C incubation period (from 0.5 to 30 min), the reactions were stopped by treatment with 0.2% SDS (final concentration) followed by proteinase K treatment (50 μg/reaction sample). DNA purified by chloroform-phenol extraction and ethanol precipitation was digested with NdeI and EcoRV. The digestion products were separated by 1% agarose gel electrophoresis, and DNA bands were visualized by ethidium bromide staining.

Strand cleavage and strand joining reactions were carried out in the recombination buffer with 0.05 pmol of the respective ^32^P-labeled half-site substrates per reaction and Flp or Flp-SUMO ranging from 0.2 pmol to 2 pmol. At the end of 30 min incubation at 30°C, reactions were stopped by adding 0.2% SDS, and processed without proteinase K treatment. The cleavage and joining reactions were analyzed by electrophoresis in 12% SDS-polyacrylamide (29:1 crosslinking) and 12% polyacrylamide-urea (19:1 crosslinking) gels, followed by phosphorimaging or autoradiography.

### Strand cleavage by a complementing pair of R191A and Y343F mutants; strand joining by a Y343F mutant in the presence of an R191A mutant

First, tubes were set up in pairs on ice with one tube within a pair containing the ^32^P-labeled half-site plus the R191A mutant, and the other containing the same labeled half-site plus the Y343F mutant. The amounts of half-site and protein in each tube were 0.05 pmol and 0.5 pmol, respectively, in 15 μl of 1.5x recombination buffer. Following 10 min on ice to allow full occupancy of the half-site by protein, 7.5 μl each of the binding mixture were withdrawn from each set of paired tubes, and transferred simultaneously to fresh tubes (maintained at 30°C) containing 2.5 pmol of an unlabeled DNA fragment with one *FRT* site in 15 μl 0.5x recombination buffer. The contents were gently mixed in each tube and incubated for 30 min. Except for the difference in the substrate half-sites, the strand cleavage and joining reactions were similar in other respects. The reactions were analyzed by SDS-polyacrylamide gel (12%) electrophoresis (for cleavage) and by polyacrylamide-urea gel (12%) electrophoresis (for joining).

### Detection and quantitation of DNA and protein bands

Radioactively labeled DNA bands, captured on a phosphor storage screen (Bio-Rad), were scanned using a Typhoon Trio Phosphorimager (GE-Healthcare). Unlabeled DNA bands were visualized in agarose gels by ethidium bromide staining. Protein bands were detected in western blot analyses using Pierce^TM^ ECL protocol (ThermoFisher Scientific). Image analysis and quantitation of band intensities were performed using the software Quantity One (Bio-Rad; version 4.5.1). For recombination, strand cleavage and strand joining assays, the extent of reaction was estimated as the ratio of the intensity of product band(s) to the sum of the intensities of substrate and product bands. For DNA binding, the ratios of the bound C-I and C-II complexes to the sum of C-I, C-II and unbound DNA were determined. Protein bands were quantitated against actin as the internal control. Multiple exposures were used to compensate for large intensity differences between individual bands. Appropriate correction factors were applied, based on the linear ranges of intensity variation.

## Results

### Substitution of Flp-SUMO fusion for Flp suppresses Flp-induced cell lethality in a *siz1Δ siz2Δ* strain

Impairment in the regulation of Flp-mediated amplification leads to high 2-micron plasmid copy number [31,35,52], which induces characteristic nibbling at colony edges. This phenotype, which is more conspicuous at 20°C than at 30°C, is due to differences in plasmid copy number in individual cell lineages, resulting in variable growth inhibition and cell mortality among them. Loss of plasmid restores normal growth and smooth edges. Over time, plasmid-free [Cir^0^] cells tend to rise in the population. Thus, colony morphology and plasmid loss rates are reliable reporters of the mean plasmid load carried by cells, and indirectly of the Flp level/activity in them.

The steady-state level of sumoylated Flp in a wild type strain is ~10% of total Flp [31]. The predominant site of SUMO conjugation, mediated by Siz1 and Siz2, is Lys-375 [31] located < 50 amino acids upstream of the carboxyl-terminus (Ile-423). Replacement of Lys-375 by arginine partially recapitulates the effects of *siz1Δ siz2Δ* in a wild type background, yielding ~4-fold increase in Flp, ~2-fold higher plasmid copy number, and consistently more abnormal colonies on plates incubated at 20°C [31]. Given the relative proximity of Lys-375 and Ile-423, we suspected that Flp containing the SUMO moiety (amino acids 1–96) as a carboxyl-terminal extension is likely to functionally mimic Flp(K375-SUMO). If so, Flp-SUMO may justifiably be utilized as a surrogate for Flp(K375-SUMO) in addressing potential differences between native and modified Flp in their relative stability *in vivo* as well as their DNA recognition and catalytic properties *in vivo* and/or *in vitro*. It is nearly impossible to study exclusively the naturally sumoylated Flp in a cell, as it would be diluted out by the excess unmodified version. In addition, the extent of the modification (~10%) makes it technically quite challenging to obtain sufficient quantities of Flp(K375-SUMO) for *in vitro* analyses.

In order to test whether Flp-SUMO can redress the effects of *siz1Δ siz2Δ* on colony morphology, we transformed [Cir^0^] strains with 2-micron plasmid derivatives engineered to express Flp or Flp-SUMO under the control of the native *FLP*-promoter (S1 Fig). Except for manipulations of the *FLP* locus and an insertion of the *ADE2* marker, the reporter plasmids retained the overall organization of the native 2-micron plasmid genome. Note that the expressed Flp, Flp-SUMO and their variants contained the HA-His8 epitope tag at their carboxyl-termini. For simplicity, these proteins as well as the plasmids expressing them are referred to without mentioning the tag.

**Fig 2:**
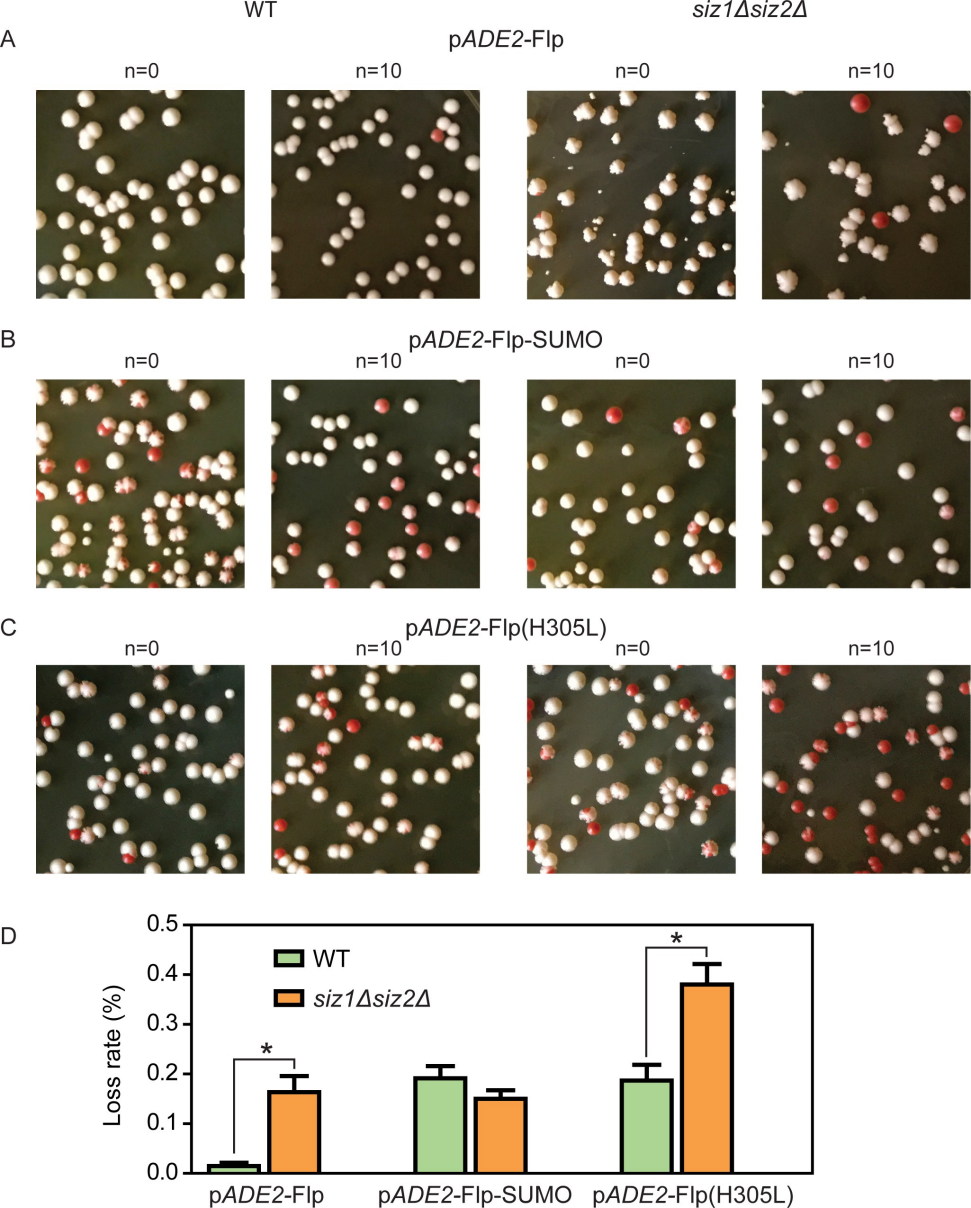
Colony morphology in the presence of plasmids expressing Flp, Flp-SUMO or Flp(H305L) and plasmid stability. The 2-micron plasmid derivatives p*ADE2*-Flp (**A**), p*ADE2*-Flp-SUMO (**B**) and pADE2-Flp(H305L) (**C**) are schematically illustrated in S1 Fig, and their relevant features described in the legend. The start of non-selective growth in YEPD liquid medium, following overnight growth in medium lacking adenine, is indicated by n = 0. Additional growth for ten generations is indicated by n = 10. The colony morphologies shown here (**A**-**C**) (see also S3 Table) are from YEPD plates incubated at 26°C for 5 days. Only white colonies (composed of plasmid containing cells), or fully white patches in colonies with red sectors, were scored for nibbling. Red colonies resulting from plasmid loss were omitted. Magnified views of representative normal and aberrant colonies are shown above S3 Table The plasmid loss rate per generation (**D**) was calculated from colonies incubated for 3–4 days at 30°C. An asterisk indicates p < 0.05 by the two-tailed test for significance. ‘WT’ denotes the wild type strain (in other figures as well).

The large fraction of nibbled colonies (> 75% at 26°C) in the *siz1Δ siz2Δ* strain containing p*ADE2*-Flp was substantially reduced (~1%) when the strain harbored p*ADE2*-Flp-SUMO (Fig 2A and 2B; S3 Table). The enlarged images of colonies shown above S3 Table highlight the difference between smooth and nibbled edges. The frequency of mini-colonies in the population, signifying highly retarded cell growth or extensive cell death, also showed a corresponding reduction (from ~18% to ~3%) (S3 Table). The nibbled- and mini-colony phenotypes were consistent with a ~5-fold increase in the mean copy number of p*ADE2*-Flp in *siz1Δ siz2Δ* compared to the wild type (S2 Fig). For comparison, the increase in the native 2-micron plasmid copy number in the mutant strain was ~10-fold (S2 Fig). As Flp-SUMO is recombination-competent (S3 Fig), the lack of nibbling in *siz1Δ siz2Δ* harboring p*ADE2*-Flp-SUMO was not due to Flp-SUMO being inactive in generating *FRT*-nicks, which are intermediates in the recombination reaction (and potential initiators of BIR).

The catalytic variant Flp(H305L) expressed by p*ADE2*-Flp(H305L) (S1 Fig) is strongly defective in strand joining *in vitro* and *in vivo* in yeast [53–55], and is expected to cause an accumulation of the *FRT*-nicked intermediate. The variant is inactive in recombination. The presence of p*ADE2*-Flp(H305L), contrary to expectation, produced few nibbled (1–2%) or mini-colonies (~3%) either in the wild type or in the *siz1Δ siz2Δ* strain at 26°C (Fig 2C; S3 Table). Presumably, sumoylation of Flp(H305L) at Lys-375 in the wild type strain was sufficient to suppress excessive *FRT*-nicking. The lack of nibbling even in the *siz1Δ siz2Δ* strain was likely due to high p*ADE2*-Flp(H305L) missegregation, signified by the relative abundance of red (*ade2*) and red-sectored colonies (Fig 2C) (see also plasmid loss rates in Fig 2D). As a result, mother cultures (n = 0 in Fig 2) would be enriched in cells with low copy numbers of p*ADE2*-Flp-SUMO as well as plasmid-free cells capable of resuming growth when provided with adenine. Such cells would be further enriched during non-selective growth (from n = 0 to n = 10) due to their fitness advantage. In fact, overexpression of Flp(H305L) in [Cir^+^] cells is a convenient method for rapidly curing them of the endogenous 2-micron plasmid [56].

In sum, the aberrant cell growth typical of under-sumoylation of Flp is alleviated by the expression of Flp-SUMO from the native *FLP*-promoter. The lack of anticipated nibbling in the *siz1Δ siz2Δ* strain from Flp(H305L) expression is the result of the high rate of plasmid loss induced by this Flp variant.

### Flp or Flp(H305L), but not Flp-SUMO, increases plasmid loss in a *siz1Δ siz2Δ* strain

As equal segregation of 2-micron plasmid molecules occurs in physical association with chromosomes [8–11], the high molecular weight hyper-amplified plasmid concatemers in a *siz1Δ siz2Δ* strain are likely to interfere with this process. Furthermore, the deficiency in sumoylation of Rep1 and Rep2 partitioning proteins might have an additional effect on segregation [32]. Plasmid-free cells, having higher fitness, will tend to outgrow their plasmid-containing counterparts. ‘Apparent’ plasmid stability during non-selective growth provides a reasonable test of the potential salutary effect of Flp-SUMO under conditions that proscribe normal sumoylation of Flp.

The p*ADE2*-Flp plasmid showed a significantly higher loss rate in the *siz1Δ siz2Δ* strain compared to the wild type (Fig 2D; left pair of histograms). By contrast, there was a modest improvement in the stability of p*ADE2*-Flp-SUMO in the mutant compared to the wild type (Fig 2D; middle pair of histograms). The lower basal stability of p*ADE2*-Flp-SUMO than p*ADE2*-Flp in the wild type strain (Fig 2D; green histograms of the left and middle pairs) might result from the larger size of p*ADE2*-Flp-SUMO, the particular modification of the *FLP* locus, or from potential additional sumoylation at Lys-375 (which would be ameliorated by *siz1Δ siz2Δ*). Consistent with the expected increase in steady state DNA damage at *FRT* and the attendant plasmid hyper-amplification, p*ADE2*-Flp(H305L) was more unstable than p*ADE2*-Flp in the wild type strain (Fig 2D; green histograms of the left and right pairs). The instability of p*ADE2*-Flp(H305L) was worsened by *siz1Δ siz2Δ* (Fig 2D; right pair of histograms), suggesting that Flp(H305L), analogous to Flp, is also downregulated via sumoylation.

The comparable loss rates of p*ADE2*-Flp(H305L) and p*ADE2*-Flp in the wild type and the *siz1Δ siz2Δ* strains, respectively, (Fig 2D; orange histogram of the left pair and green histogram of the right pair) might appear to suggest that normal sumoylation of Flp(H305L) and strongly reduced sumoylation of Flp are more or less equivalent with respect to the amount of strand nicks that the two proteins produce at *FRT*. However, unlike Flp, Flp(H305L) cannot resolve an amplified plasmid concatemer by recombination, nor can it counter plasmid missegregation by recombination-mediated amplification (Fig 1A and 1B). These factors may aggravate the instability of p*ADE2*-Flp(H305L) in the *siz1Δ siz2Δ* strain.

The plasmid stability results demonstrate that the increased formation and expansion of plasmid-free cells triggered by *FRT*-nicks in a *siz1Δ siz2Δ* host is strongly suppressed when this strain expresses Flp-SUMO instead of Flp or Flp(H305L).

### Cell lethality caused by Flp(H305L) in conjunction with *mus81Δ* or *yen1Δ* is efficiently reversed by replacement of Flp(H305L) with Flp(H305L)-SUMO

**Fig 3:**
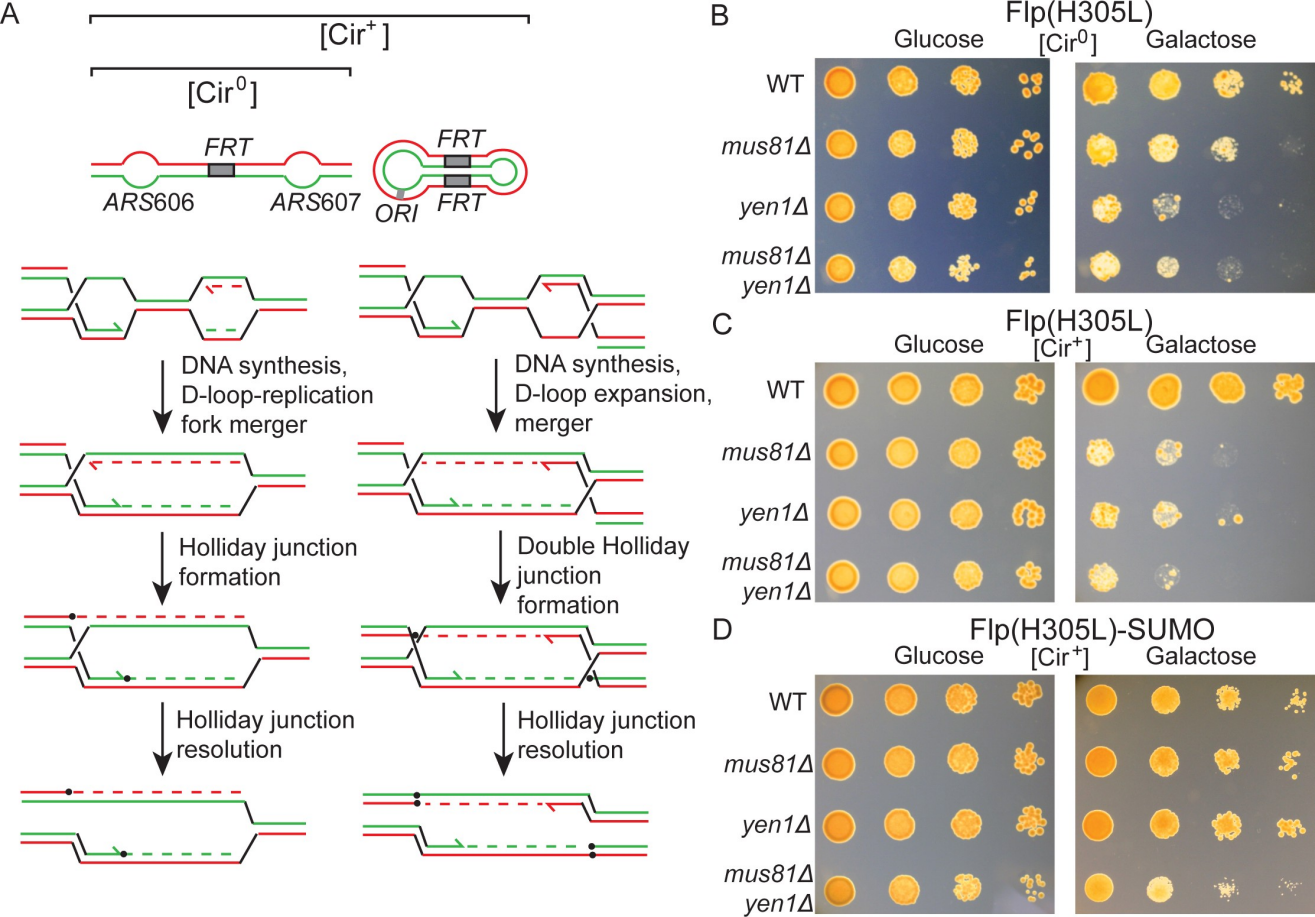
Flp(H305L)-SUMO, in contrast to Flp(H305L), mollifies the requirement for Mus81 and Yen1 in cells containing chromosomal plus extra-chromosomal *FRT* sites. **A**. The single *FRT* site inserted into chromosome VI between replication origins *ARS*606 and *ARS*607 [53] is indicated at the top left. In the [Cir^0^] strain, this is the only target for strand cleavage by Flp(H305L) or Flp(H305L)-SUMO. Additional *FRT* targets are presented by multiple copies of the 2-micron plasmid in the [Cir^+^] strain (top right). The schematic diagrams below depict the consequences of BIR triggered by strand nicks at *FRT* coupled to DNA replication. The merger of an expanding D-loop with an oncoming replication fork (left) will result in a repair intermediate with a DNA branch at one end. The encounter between two expanding BIR-induced D-loops (right) will generate an intermediate with DNA branches at both ends. Each of these branches can mature into a Holliday junction via strand ligation. Resolution of these intermediates may be performed by Yen1 or Mus81-Mms4 or both. **B**-**D**. Serial dilutions of [Cir^0^] or [Cir^+^] cells, uninduced (glucose) or induced (galactose) for the expression of Flp(H305L) or Flp(H305L)-SUMO, were spotted on YEPD plates, and growth was examined after 3-day incubation at 30°C. The inoculum at the left-most spots was ~5 x 10^4^ cells, with the spots to their right representing sequential 10-fold dilutions.

The BIR pathway promotes the repair of one-ended double strand breaks—such as those resulting from the arrival of a replication fork at a Flp-nicked *FRT* site [31,33,39,57,58]. Consistent with the mechanism diagrammed in Fig 1C, the formation of amplified high molecular weight plasmid DNA is dependent on strand cleavage by Flp, and requires Pol32 as well as Rad proteins involved in BIR [33]. The initial D-loop intermediate of BIR formed by strand invasion of homologous DNA may be processed into a replication fork. Alternatively, it may mature into a Holliday junction by convergence with a replication fork from the opposite direction (Fig 3A; left). The coalescence of two D-loops expanding in opposite directions would generate a double Holliday junction (Fig 3A; right). The organization of the two *FRT* sites and the location of the bi-directional replication origin within the 2-micron plasmid provide opportunities for a Flp-mediated BIR D-loop to meet a replication fork, or a second such D-loop, approaching it head-on (Fig 3A). As the Flp-induced single strand nicks may occur at one or both of the plasmid *FRT* sites, and on either DNA strand within a site, head-to-tail configuration of two D-loops or a D-loop and a replication fork is also possible. Crystal structures and *in vitro* experiments rule out double strand breaks at *FRT* by Flp [50,59–61], minimizing the probability of two-ended double strand break repair in *FRT* DNA damage.

The resolution of specialized DNA structures such as Holliday junctions and D-loops in yeast require Yen1 and/or Mus81-Mms4 activities [53]. Induction of Flp(H305L) in a strain containing an *FRT* site inserted between two strong replication origins in a chromosome results in poor viability in the *yen1Δ mus81Δ* background [53]. We utilized isogenic [Cir^0^] and [Cir^+^] strains harboring an identical chromosomal insertion of *FRT* to test whether the presence of additional copies of plasmid-borne *FRT* sites would aggravate the Flp(H305L)—*yen1Δ* or the Flp(H305L)-*mus81Δ* effect, and whether cell survival can be improved by replacing Flp(H305L) by Flp(H305L)-SUMO.

In the [Cir^0^] strain, *mus81Δ* caused a decrease in colony forming units upon Flp(H305L) induction, with *yen1Δ* and *yen1Δ mus81Δ* displaying a stronger effect (Fig 3B). The loss of viable colonies from each single mutation as well as the double mutation was magnified in the [Cir^+^] strain (Fig 3C). Expression of Flp(H305L)-SUMO in place of Flp(H305L) restored cell survival in the single mutants to nearly the same level as in the wild type (Fig 3D). The palliative response to Flp(H305L)-SUMO, though not as strong, was evident in the double mutant as well (Fig 3D).

In the absence of Mus81 or Yen1 or both, the branched intermediates of Flp(H305L)-induced BIR (three or four-way DNA junctions) appear to accumulate, to the detriment of the cell. The apparent reduction of these intermediates in the presence of Flp(H305L)-SUMO is consistent with an abatement in the formation of unsealed strand nicks at *FRT* that precedes the BIR events. Furthermore, these results corroborate the previous inference that the DNA damage induced by Flp(H305L) at plasmid *FRT* sites may be masked by accelerated plasmid loss from cells.

### The p*ADE2*-Flp-SUMO plasmid causes cell lethality, and is destabilized, in the absence of Slx5 or Slx8

The Slx5-Slx8 STUbL (sumo targeted ubiquitin ligase) regulates a wide range of cellular functions in yeast that include gene expression, quality control of nuclear proteins, DNA damage repair, and chromosome stability [62–66]. The coupling to ubiquitin-proteasome systems *via* Slx5-Slx8-mediated recognition of conjugated SUMO, or native surface features that mimic SUMO, may bring about not only the degradation of particular target proteins but also the functional re-localization of multi-subunit protein machines. Examples include proteolysis of the Mot1 transcription factor [67] or the Matα2 repressor [68], and the relocation of double strand DNA breaks in G1 cells to repair centers stationed at the nuclear periphery [69]. Ubiquitin-dependent proteolysis of sumoylated Flp appears to be one important mechanism for the post-translational regulation of Flp. The levels of Flp, including its SUMO-conjugated form, are increased in *slx5Δ* and *slx8Δ* strains [33]. We therefore tested whether the beneficial effects of Flp-SUMO observed in the *siz1Δ siz2Δ* strain would be reversed in an *slx5Δ* or *slx8Δ* mutant.

**Fig 4:**
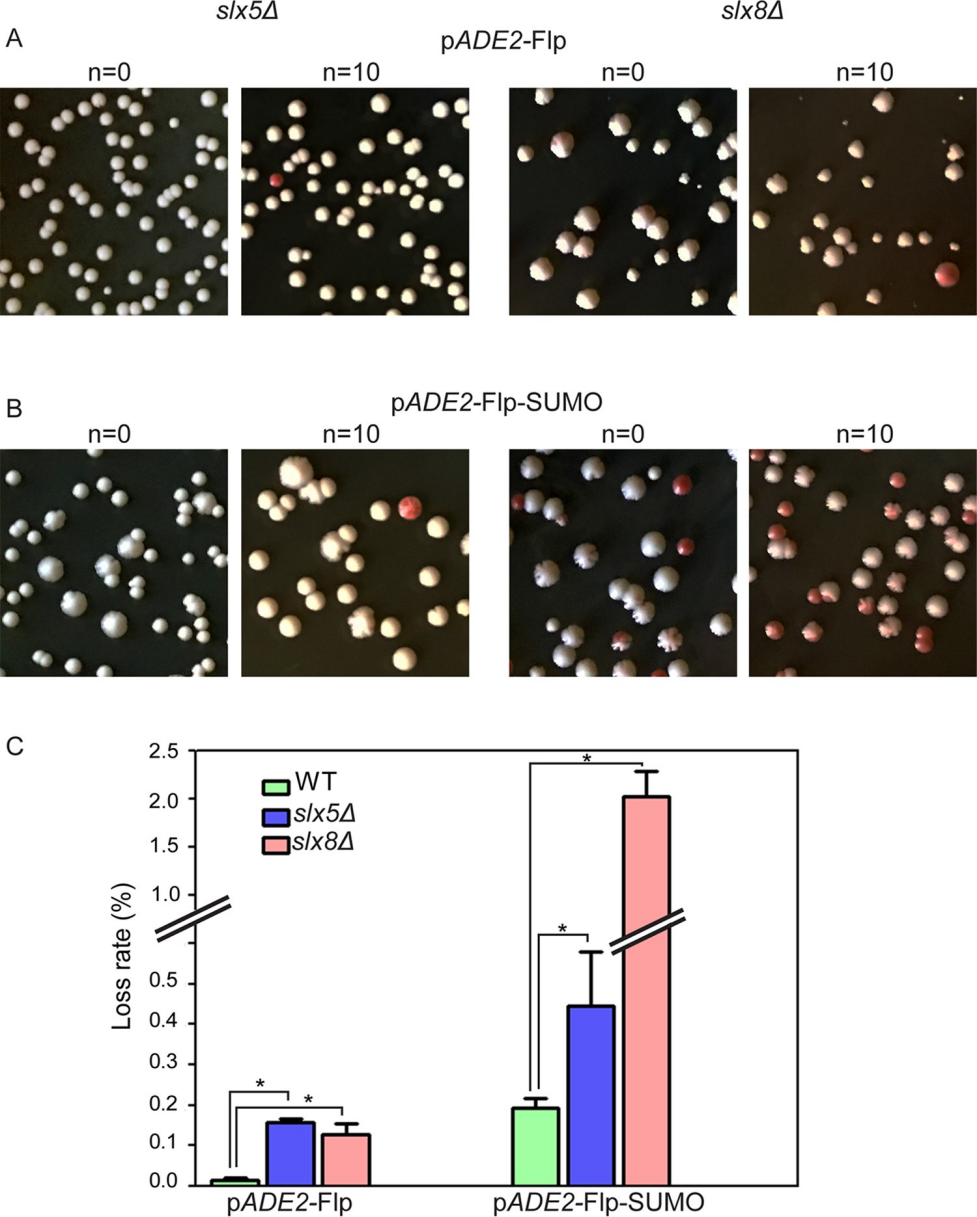
The p*ADE2*-Flp-SUMO plasmid fails to prevent nibbled colonies and increased frequency of plasmid-free cells in *silx5Δ* and *slx8Δ* strains. The protocols for scoring colony phenotypes (**A**, **B**) and estimating plasmid loss rates (**C**) were similar to those outlined under Fig 2. The cells seeded on plates at n = 0 and n = 10 were grown at 26°C for 5 days for scoring nibbled and mini-colonies (see S3 Table). Values for plasmid loss were derived from colonies on plates incubated at 30°C, and are directly comparable to those in Fig 2D. An asterisk denotes p < 0.05.

The *slx5Δ* and *slx8Δ* strains containing p*ADE2*-Flp or p*ADE2*-Flp-SUMO (Fig 4A and 4B; S3 Table) formed nibbled colonies in contrast to the wild type strain containing either plasmid and the *siz1Δ siz2Δ* strain containing p*ADE2*-Flp-SUMO (Fig 2A and 2B; S3 Table). The fraction of mini-colonies caused by p*ADE2*-Flp was also elevated in the *slx8Δ* mutant compared to the wild type (S3 Table). Mini-colonies induced by p*ADE2*-Flp-SUMO in the *slx5Δ* and *slx8Δ* strains were only slightly more than those in the wild type or the *siz1Δ siz2Δ* mutant (S3 Table).

The loss of p*ADE2*-Flp-SUMO was more rapid in the *slx5Δ* and *slx8Δ* strains than in the wild type (Fig 4C) or the *siz1Δ siz2Δ* (Fig 2D) strains. The *slx5Δ* or *slx8Δ* mutation caused instability of p*ADE2*-Flp as well (Fig 4C), the plasmid loss rates being comparable to that in the *siz1Δ siz2Δ* strain (Fig 2D).

A key point underscored by these findings is that both *slx5Δ* and *slx8Δ*, but not *siz1Δ siz2Δ*, display the phenotypes of aberrant p*ADE2*-Flp-SUMO amplification (Figs 2D and 4C; S3 Table). Furthermore, *slx8Δ* phenocopies *siz1Δ siz2Δ* closely when they contain p*ADE2*-Flp (Figs 2D and 4C; S3 Table). The trend is similar for *slx5Δ* albeit less striking than *slx8Δ*. Some of the quantitative differences among individual mutants may be reconciled by the involvement of the ubiquitin and SUMO pathways in the pleiotropic regulation of a variety of cellular processes.

**Fig 5:**
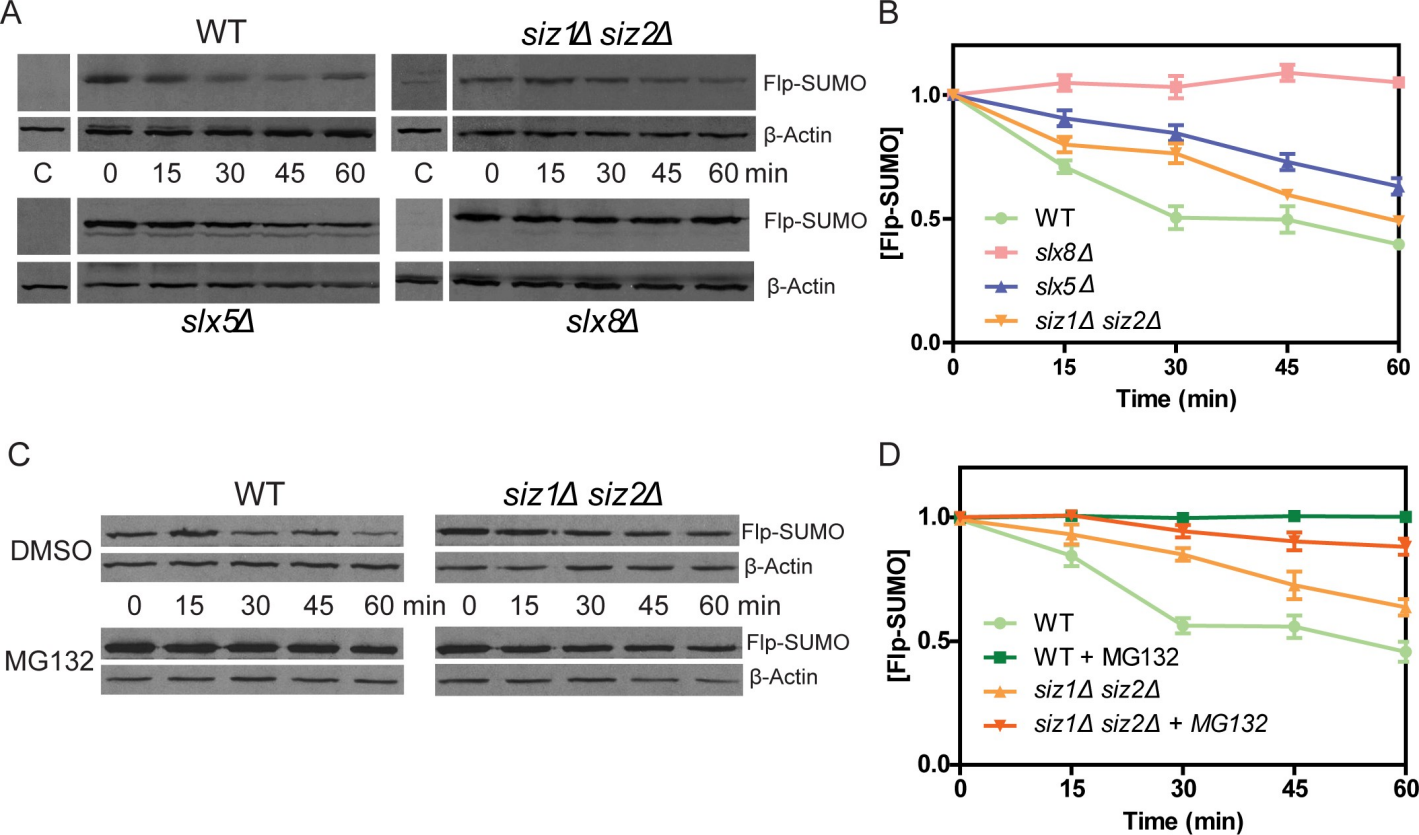
Turnover of Flp-SUMO is slowed down by *siz1Δ siz2Δ*, *slx5Δ*, *slx8Δ* or by proteasome inhibition. **A**, **B**. Flp-SUMO was expressed from a *GAL* promoter controlled expression cassette inserted into chromosome IV at the *TRP1* locus. Following galactose induction for 2 hr, protein synthesis was inhibited by cycloheximide (0 min), and the levels of Flp-SUMO were followed over a 60 min time course by western blot analysis. The lanes marked ‘C’ refer to uninduced cells, incubated in glucose medium for 2 hr. Band intensities were plotted after normalizing them against those for actin as a control. The relative intensity at 0 min was assigned a value of 1.0. Flp-SUMO was visualized in the western blots using an antibody to the HA-tag present at the carboxyl-terminus. Actin was detected using an antibody to the native protein. **C**, **D**. The analyses were similar to those in **A** and **B**, except that the culture conditions were slightly modified (Materials and Methods), and the proteasome inhibitor MG-132 was added to the experimental samples at 90 min into induction (30 min prior to time 0).

The phenotypic responses of the mutants to p*ADE2*-Flp or p*ADE2*-Flp-SUMO suggest that normal regulation of Flp requires sumoylated Flp to be ubiquitinated and channeled to the proteasome for degradation. Furthermore, SUMO conjugated physiologically by Siz1-Siz2 (principally to Lys-375) within Flp and SUMO attached artificially to the Flp carboxyl-terminus are functionally interchangeable in recognition by the Slx5-Slx8 STUbL. As a result, Flp becomes deleterious in *siz1Δ siz2Δ*, *slx5Δ* and *slx8Δ*; Flp-SUMO is harmful only in the latter two. Corroborating these interpretations, the half-life of Flp-SUMO was higher in *slx5Δ* compared to that in the wild type or the *siz1Δ siz2Δ* mutant, while Flp-SUMO degradation was almost completely blocked by *slx8Δ* (Fig 5A and 5B). The turnover of Flp-SUMO in the wild type and *siz1Δ siz2Δ* was suppressed by the proteasome inhibitor MG-132 (Fig 5C and 5D).

### Flp-SUMO has weaker recombinase activity than Flp *in vitro*

In principle, sumoylation may not only control the steady state levels of Flp *in vivo* but may also modulate its recognition of the *FRT* site and/or its active site functions. DNA damage and resultant BIR in the 2-micron plasmid may be curtailed by preferential exclusion of sumoylated Flp from *FRT* sites, reduced strand cleavage and/or enhanced strand joining by the modified protein, or by combinations of these attributes. In order to probe the effects of sumoylation on Flp activities, we utilized *in vitro* assays using purified Flp and Flp-SUMO. Based on their very similar *in vivo* responses to multiple mutations in the SUMO/ubiquitin pathways, Flp-SUMO is presumed to be an authentic substitute for Flp(K375-SUMO) *in vitro* as well. This premise is also supported by structural considerations. In the Flp recombination/synaptic complexes [59,70], the distances from Lys-375 or the carboxyl-terminus (Ile-423) to key active site residues are nearly the same, the maximum difference being ~4 Å (S4 Table). As such, potential structure-function perturbations from the SUMO moiety are not expected to be different between Flp sumoylated at Lys-375 and Flp-SUMO.

**Fig 6:**
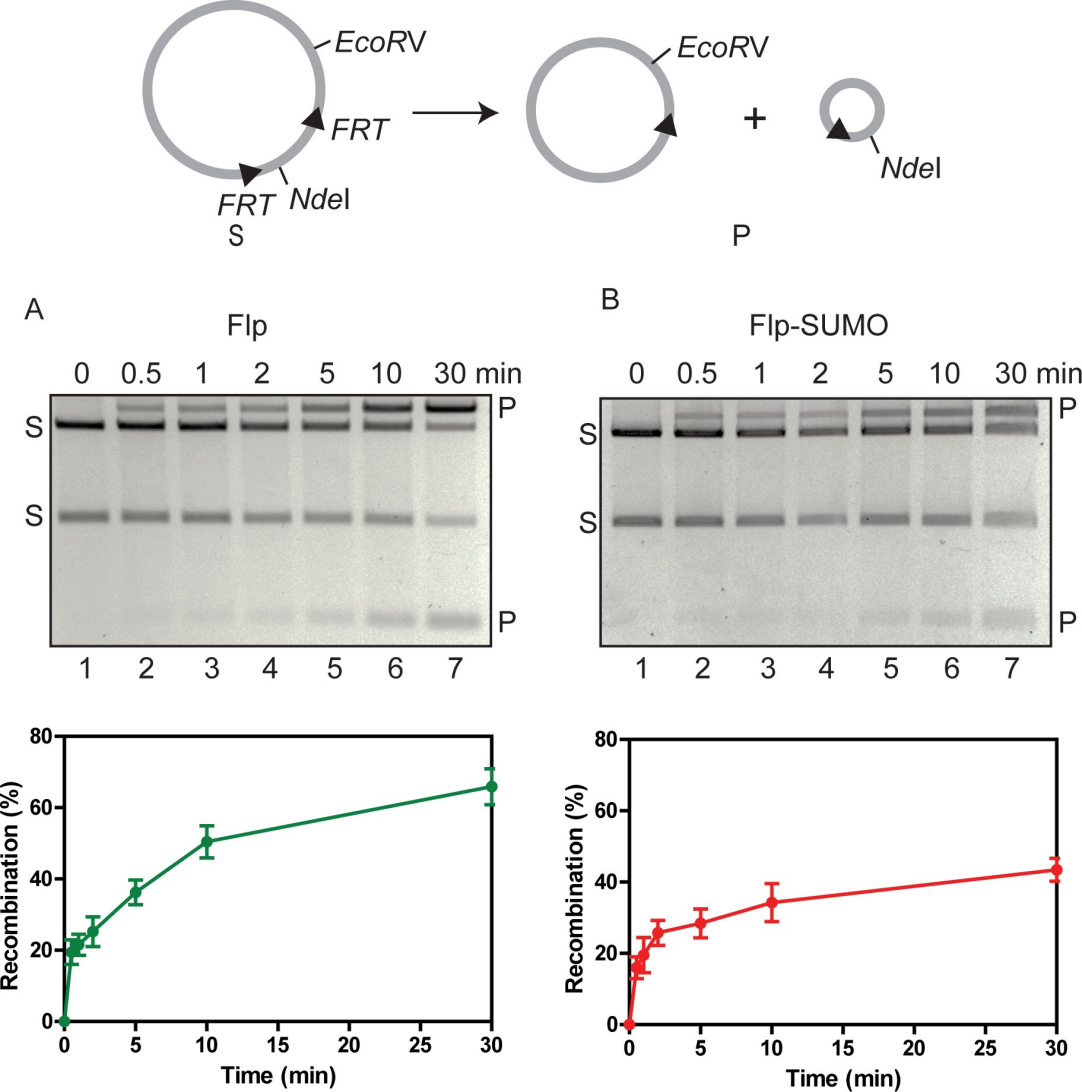
Flp-SUMO is less active than Flp in recombination between head-to-tail *FRT* sites placed within a circular plasmid. **A**, **B**. The recombination reaction is schematically outlined at the top. The DNA fragments generated by EcoRV plus NdeI digestion of the substrate plasmid (labeled as ‘S’) and the recombinant products (labeled as ‘P’) were distinguished by agarose gel electrophoresis. The assays, performed at 30°C, utilized 0.2 pmol of the substrate plasmid (0.8 pmol binding site for monomeric Flp) and 1.0 pmol of Flp or Flp-SUMO (containing carboxyl-terminal HA-His8 tag). As shown in S4 Fig, there was no measurable difference between native Flp and tagged Flp in recombination activity.

In a time-course deletion reaction between two head-to-tail *FRT* sites, Flp-SUMO was approximately 60% as active as Flp (Fig 6A and 6B). To be consistent with *in vivo* experiments, the *in vitro* analyses were performed using proteins containing the HA-His8 tag at the carboxyl-terminus. Native Flp and the HA-HIS8-tagged variant were nearly identical in recombination activities (S4 Fig).

The contra-effect of the SUMO moiety on recombination may occur during Flp-*FRT* association and assembly of the recombination complex, or within a pre-assembled complex. Allosteric interactions and collaborative assembly of the strand cleavage active site between neighboring *FRT*-bound Flp monomers are key features of the recombination reaction, which is carried out within a synaptic structure containing four Flp monomers bound to two partner *FRT* sites [50,59]. Thus, sumoylation of just one Flp monomer *in vivo*, out of two bound to an *FRT* site or four bound to a pair of synapsed *FRT* sites, may protect against DNA damage at *FRT* by attenuating Flp activity. The role of monomer-monomer interactions in Flp catalysis is dealt with in more detail in the context of the strand cleavage and strand joining activities of Flp (see below).

### Flp has higher *FRT* binding efficiency and binding cooperativity than Flp-SUMO

The weaker recombinase activity of Flp-SUMO than Flp could, at last in part, be due to its impaired recognition of the *FRT* site. Flp binds as a monomer to each of the two 13 bp head-to-head binding elements flanking the 8 bp strand exchange region of a minimal *FRT* site, and cooperative interactions between the bound monomers stabilize the *FRT*-Flp dimer complex. We tested Flp and Flp-SUMO for differences in the formation of singly or doubly bound complexes with a DNA fragment containing one copy of the *FRT* site. These two complexes were resolved by their electrophoretic mobility in native polyacrylamide gels.

**Fig 7:**
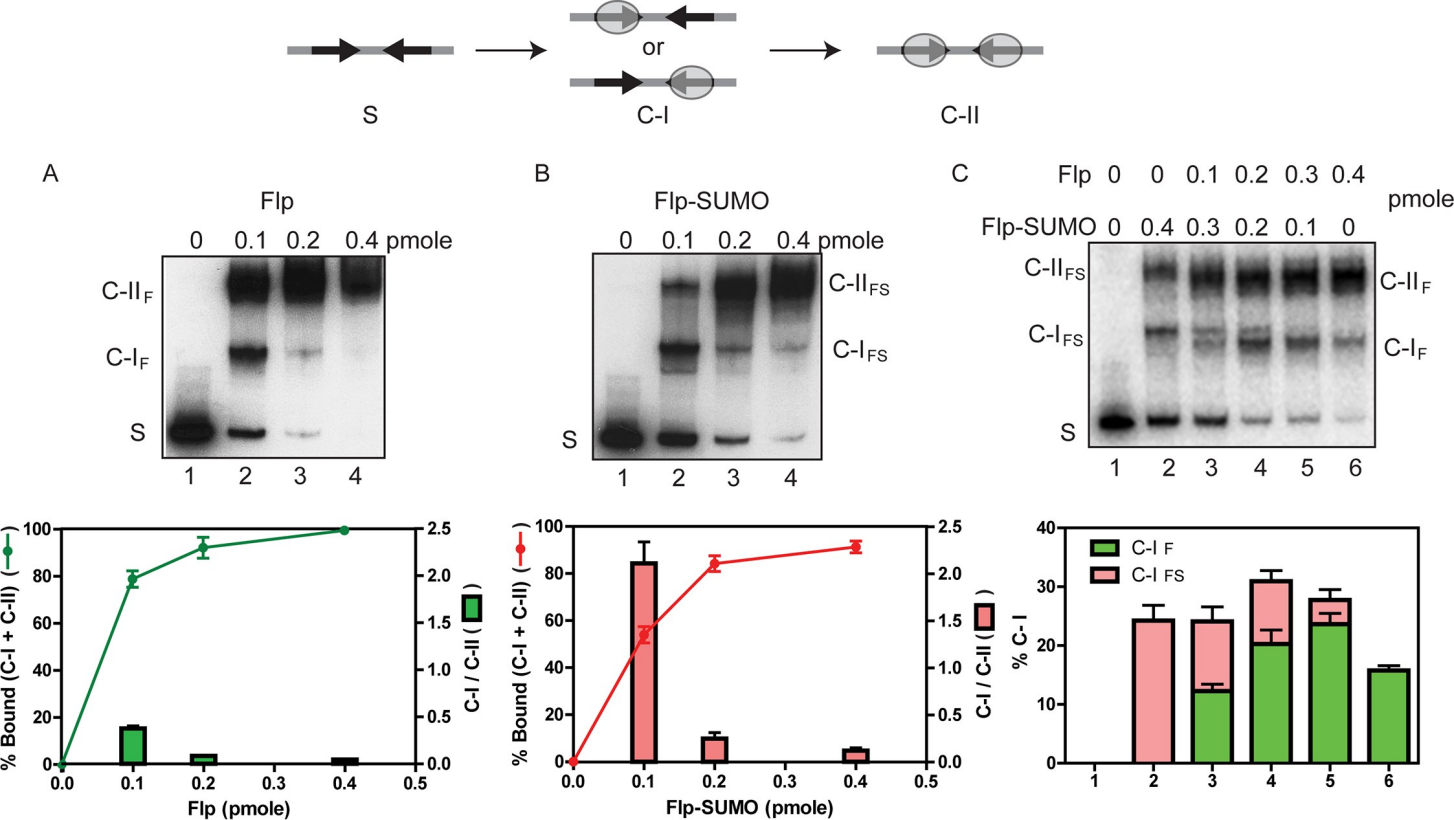
*FRT*-binding is weakened in Flp-SUMO. **A**-**C**. Stepwise association of individual Flp monomers to the two binding elements (arrows oriented head-to-head) of an *FRT* site is diagrammed schematically at the top. The binding reactions contained 0.05 pmol of the DNA containing one *FRT* site (labeled as ‘S’), equivalent to 0.1 pmol Flp-binding element, and the indicated amounts of Flp or Flp-SUMO. The binding mixtures were incubated on ice for 20 min, and the unbound and bound DNA were fractionated by electrophoresis in native 5% polyacrylamide gels. The DNA-protein complexes corresponding to the occupancy of one binding element and both binding elements are called C-I and C-II, respectively. The subscripts ‘F’ and ‘FS’ refer to Flp and Flp-SUMO, respectively.

At identical molar ratios of protein per binding element, the combined amount of monomeric (C-I) and dimeric (C-II) complexes formed were lower for Flp-SUMO than Flp (Fig 7A and 7B). It took approximately twice as much Flp-SUMO as Flp to convert the same amount of *FRT*-DNA into the bound form. Furthermore, there was a striking difference in binding cooperativity between Flp-SUMO and Flp. At 1:1 molar ratio of protein to binding element (lane 2 in Fig 7A), C-II formation by Flp was favored over C-I by a factor of ~3. By contrast, Flp-SUMO formed ~2-fold more C-I than C-II under the same condition (lane 2, Fig 7B).

The disparity in *FRT* binding and cooperativity between Flp and Flp-SUMO was also highlighted by binding reactions containing a mixture of the two proteins (Fig 7C). Even at 1:3 molar ratio, Flp surpassed Flp-SUMO in *FRT* occupancy, as indicated by the strong reduction of the C-II complex formed by Flp-SUMO and the appearance of the Flp C-I and C-II complexes (lane 3, Fig 7C). At this ratio, the C-I complexes formed by the two proteins were roughly equal. The plot of C-I ratios underestimates the advantage of Flp over Flp-SUMO in *FRT* binding as it does not capture the much stronger cooperativity of Flp in C-I → C-II conversion (Fig 7A and 7B). The band corresponding to the heterodimeric C-II complex, expected to migrate just above the Flp C-II complex, was not well resolved from it. At equimolar or higher Flp to Flp-SUMO ratios (lanes 4–6, Fig 7C), the C-I and C-II complexes were predominantly the Flp-bound forms.

Extrapolation of the *in vitro* DNA binding results to the *in vivo* situation suggests that sumoylation of Flp would diminish its binding to the 2-micron plasmid *FRT* sites. In addition, or conversely, sumoylation of *FRT*-bound Flp might accelerate the dissociation of the modified protein from DNA. Either or both of these mechanisms would provide a safeguard against potentially detrimental strand cleavage events at *FRT*.

### Flp-SUMO is attenuated in strand cleavage

The strongly conserved catalytic hexad cluster of the tyrosine site-specific recombinase family (to which Flp belongs), including the invariant tyrosine nucleophile, is represented in Flp by Arg-191, Lys-223, His-305, Arg-308, Trp-330 and Tyr-343. In the shared active site for strand cleavage, Tyr-343 from one Flp monomer is donated to the pro-active site of the second neighboring Flp monomer, which provides the other five catalytic residues [50,59]. Strand joining in the cleaved intermediate, harboring a 3’-O-phosphotyrosyl bond, is promoted by one Flp monomer utilizing the 5’-hydroxyl group from DNA as the nucleophile [50,71]. In order to appraise whether sumoylation affects the chemical competence of the Flp active site, we assayed Flp and Flp-SUMO in strand cleavage and strand joining reactions *in vitro*.

Reactions were performed using half-site substrates containing a single Flp binding element and one scissile phosphate [71–74]. Interactions between two Flp monomers, each bound to a half-site, permits the assembly of the shared active site required for strand cleavage by the Tyr-343 nucleophile [50]. The half-sites are suitably configured, as described below, to avoid interference from the joining reaction during cleavage assays and *vice versa*.

**Fig 8:**
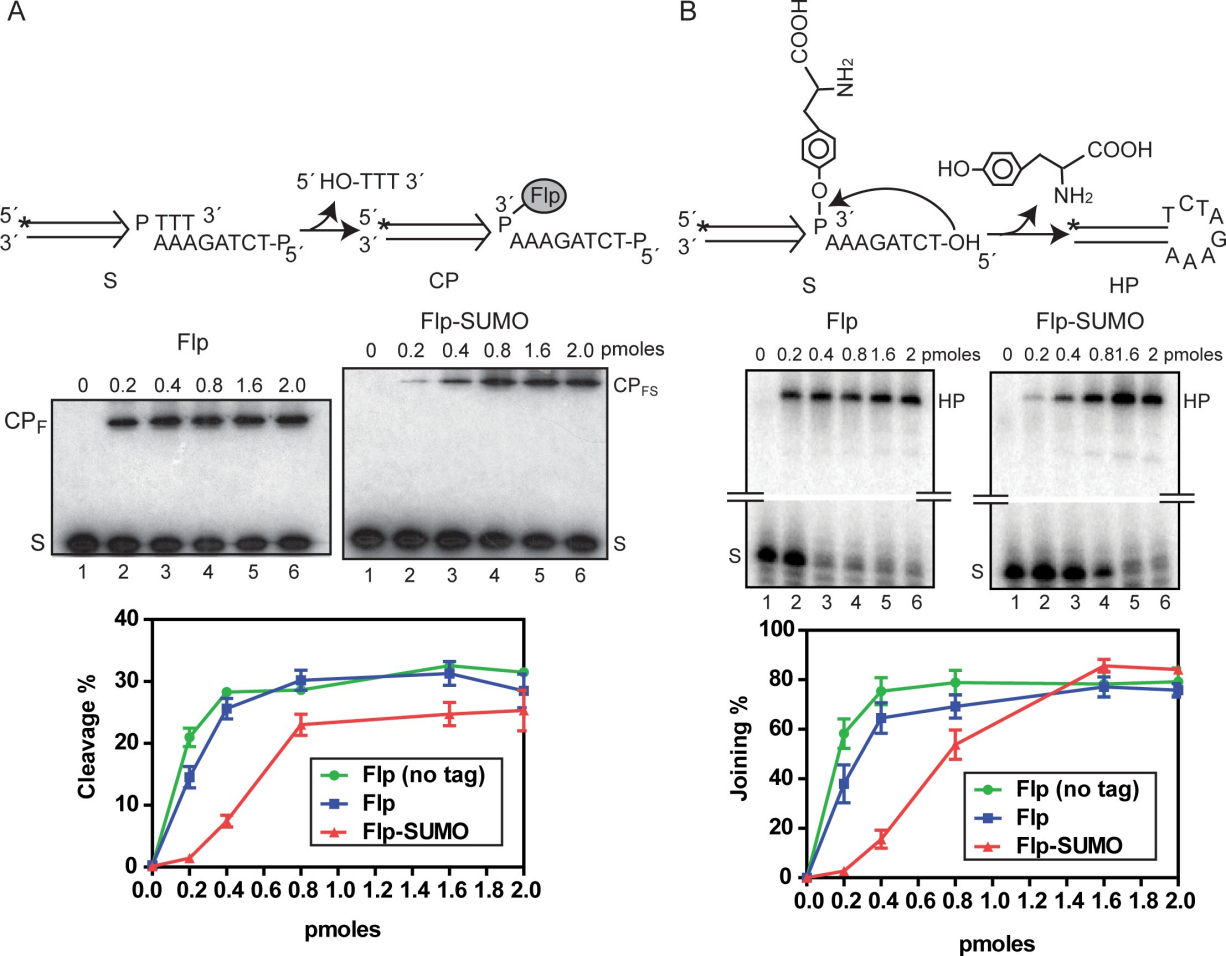
The strand cleavage and joining efficiencies are lower for Flp-SUMO than Flp. **A**. In the schematic diagram of the strand cleavage substrate (S), the single Flp binding element is signified by the parallel lines ending in an arrowhead, with ‘P’ indicating the scissile phosphate. The 5’-^32^P label on the strand containing ‘P’ is shown by the asterisk. The 5’-hydroxyl group on the complementary strand is blocked by phosphorylation. Each reaction, containing 0.05 pmol half-site plus the indicated amounts of Flp or Flp-SUMO, was incubated for 30 min at 30°C, and was analyzed by electrophoresis in 12% SDS-polyacrylamide gels. The cleavage products formed by Flp and Flp-SUMO are labeled as CP_F_ and CP_FS_, respectively. In the plots of the product yields from three separate experiments with each protein, the results with native Flp (lacking the HA-His8 tag) are also included. **B**. In the schematic diagram of the strand joining substrate (5’-^32^P-labeled; asterisk), the tyrosyl group attached to the scissile phosphate mimics the strand-cleaved recombination intermediate. The free 5’-hydroxyl group on the non-radioactive strand attacks the phosphotyrosyl bond in a ‘pseudo-joining’ reaction to give a hairpin product. The reactions were performed under the conditions employed for cleavage, and were analyzed by electrophoresis in 12% denaturing polyacrylamide gels. The hairpin products formed by Flp and Flp-SUMO are identical, and are labeled as ‘HP’.

In the strand cleavage substrate, the scissile phosphate is followed by a truncated 3 nt segment from the strand exchange region (Fig 8A). The full 8 nt complement of the exchange region in the opposite strand ends in 5’-phosphate, which prevents strand joining by a hydroxyl group at this position. Furthermore, diffusion of the unstably hydrogen bonded trinucleotide product of cleavage away from the reaction center minimizes cleavage reversal.

In the strand joining substrate, the scissile phosphate is covalently linked to a tyrosyl moiety (Fig 8B) to mimic the phosphotyrosyl bridge between Flp and DNA formed during strand cleavage. The hairpin product of the joining reaction is refractory to cleavage, as it cannot support the Flp-Flp interactions required for active site assembly in *trans*.

Flp-SUMO was less active than Flp in strand cleavage as well as strand joining. The half-maximal reaction required a ~3-fold higher amount of Flp-SUMO for both steps (Fig 8A and 8B). Flp-SUMO did catch up with Flp in its V_max_ for joining, and required a ~3-fold higher protein amount. However, at a ~5-fold higher amount, Flp-SUMO was close to (but still below) saturation cleavage by Flp. Thus, the SUMO attachment reduces the catalytic activity of Flp, the adverse effect on strand cleavage being stronger than that on strand joining.

Collectively, the binding and activity assays (Figs 6–8) suggest that the differences in the catalytic efficiencies of Flp and Flp-SUMO can be accounted for primarily by reduced *FRT* binding affinity and cooperativity imposed by the modification, perhaps with some impairment of active site function in addition. The latter possibility was tested more directly by strand cleavage complementation and strand joining assays using cleavage-incompetent Flp mutants (described below).

### Flp-SUMO is less competent than Flp in the phosphate activation step of strand cleavage, but not so in the tyrosine donation step

**Fig 9:**
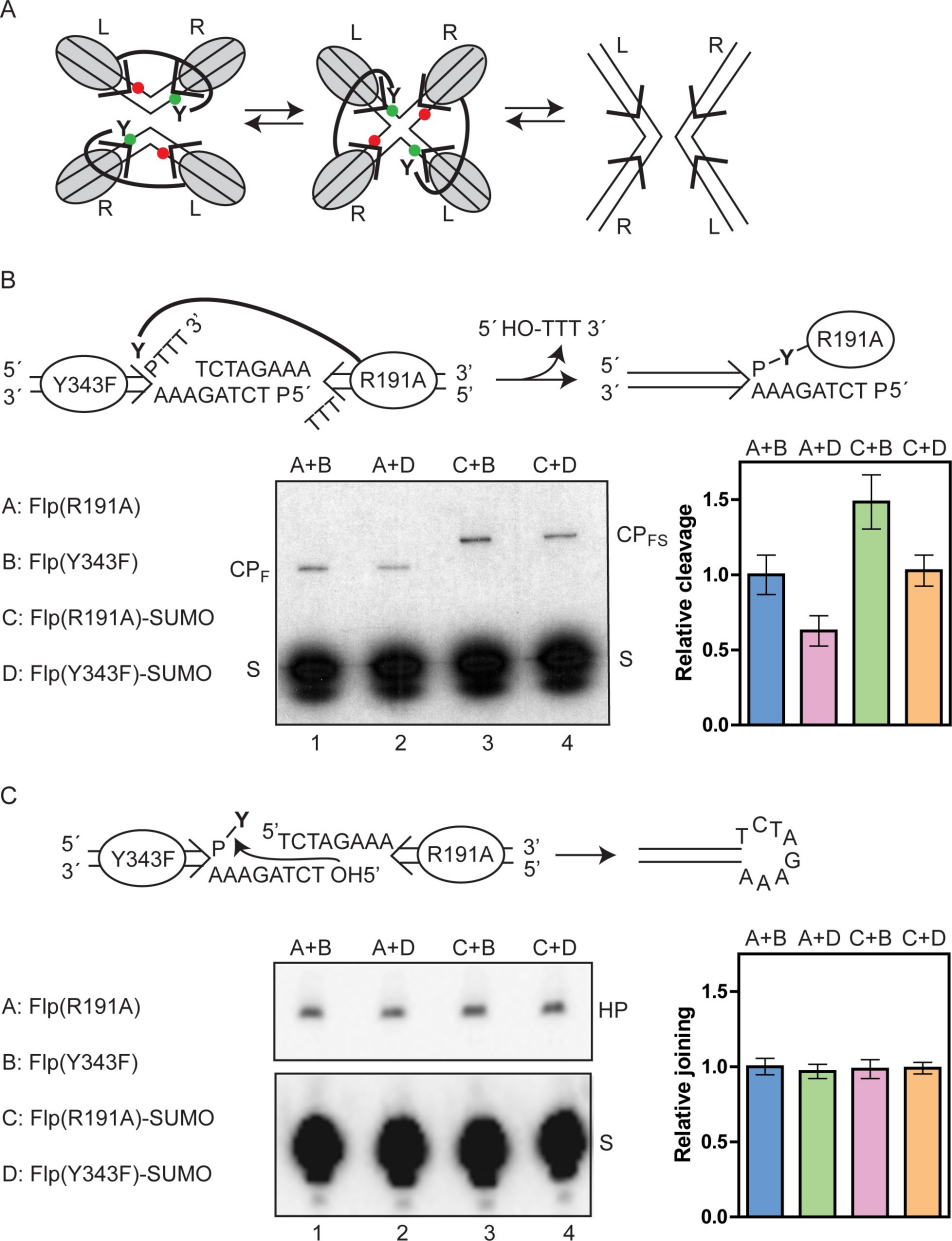
Flp-SUMO is attenuated at the phosphate activation step of strand cleavage but not at the Tyr-343 nucleophile donation step. **A**. Strand cleavage complementation by neighboring Flp monomers is schematically illustrated in the context of the recombination synapse. The four Flp monomers bound to the synapsed *FRT* sites assemble two active sites for strand cleavage at a time. Two monomers activate the scissile phosphates (green dots), to be cleaved by Tyr-343 nucleophiles received from the other two monomers. The scissile phosphates refractory to cleavage are shown as red dots. The Holliday junction formed by the first strand cleavage/exchange step isomerizes to switch the Tyr-343 donors to recipients and *vice versa*. The ensuing second strand cleavage/exchange step completes recombination. The *FRT* sites are oriented by marking them left (L) to right (R). **B**. The schematic diagram at the top shows the delivery of Tyr-343 from Flp(R191A) to cleave the half-site bound by Flp(Y343F). The indicated proteins were pre-bound separately to the 5’-^32^P-labeled half-site substrate (S), and mixed together to assay cleavage. The reactions were analyzed by SDS-polyacrylamide gel electrophoresis as described under Fig 8. The cleavage product formed by Flp(R191A) and Flp(R191A)-SUMO are indicated as CP_F_ and CP_FS_, respectively. The normalized cleavage yields from three separate assays are plotted as histograms with the output from the Flp(R191A)-Flp(Y343F) reaction as 1.0 **C**. The strand joining reaction in a half-site mediated by a bound Flp(Y343F) monomer is schematically diagrammed at top. The presence of a half-site bound by Flp(R191A) may potentially modulate the joining efficiency of Flp(Y343F) via monomer-monomer interactions. Flp(R191A) itself has no joining activity. The ^32^P-labeled half-site was bound separately by the mutants, and then mixed. The hairpin product (HP) and the unreacted substrate (S) were separated by denaturing polyacrylamide gel electrophoresis (see Fig 8). In the histogram plots representing three independent assays, the strand joining efficiency is normalized against a value of 1.0 for the Flp(Y343F) reaction containing Flp(R191A).

The two steps of single strand exchange during Flp recombination involves the assembly of the shared cleavage pocket (and *trans*-donation of Tyr-343) within individual *FRT* sites or between synapsed partner *FRT* sites [50,59] (Fig 9A). The shared active site makes it possible to catalytically complement a Flp monomer lacking Tyr-343 with one lacking a pentad residue within its pro-active site [50,71] (Fig 9B). For example, Flp(Y343F) bound to a half-*FRT* site can activate its scissile phosphate, which may then be cleaved by Tyr-343 from Flp(R191A) bound to a second half-*FRT* site. The individual mutants themselves are inactive in cleavage. We took advantage of catalytically complementing Flp and Flp-SUMO partners to ask whether Flp-SUMO is affected in the phosphate activation step or in the tyrosine donation step of strand cleavage.

The reactions were done by pre-binding the 5’-end labelled half-site separately with each one of a pair of complementing mutants, and mixing them in the presence of an excess unlabeled *FRT* to soak up unbound proteins (Fig 9B). With Flp(R191A) as the tyrosine donor, the cleavage output was higher (~1.6-fold) with Flp(Y343F) than with Flp(Y343F)-SUMO as its partner (lanes 1 and 2, Fig 9B). A similar difference in cleavage (~1.5-fold) was noted between Flp(Y343F) and Flp(Y343F)-SUMO when the tyrosine donor was Flp(R191A)-SUMO (lanes 3 and 4, Fig 9B). Flp(R191A)-SUMO was fully competent at tyrosine donation, yielding better cleavage than Flp(R191A) when partnered with Flp(Y343F) (~1.5-fold higher; lanes 1 and 3, Fig 9B) or with Flp(Y343F)-SUMO (~1.6-fold higher; lanes 2 and 4, Fig 9B). The relative cleavage efficiencies are shown as histogram plots with a value of 1.0 assigned for the Flp(R191A)-Flp(Y343F) pair.

Thus, SUMO attachment to Flp diminishes its ability to activate an adjacent scissile phosphate, and consequently causes a decrease in the probability of its cleavage. However, Flp-SUMO is more active in Tyr-343 donation for cleavage of an activated scissile phosphate. The two opposing effects would more or less cancel out. Taken together, *FRT* binding, *FRT* cleavage and cleavage complementation data suggest that weakening the cleavage potential *per se* of Flp by sumoylation is unlikely to play a significant role in protecting *FRT* against strand nicks *in vivo*. More prominent is the reduced *FRT* binding affinity and cooperativity resulting from the modification. Note that these effects would have been minimized in the complementation assays, as they utilized pre-bound half-sites.

### Flp and Flp-SUMO are equally efficient in the strand joining step

The strand joining reaction, executed by a single Flp monomer, does not require the active site Tyr-343 [72,75]. Flp and Flp(Y343F) are equally efficient in this reaction. We tested strand joining in half-site substrates pre-bound by Flp(Y343F) or Flp(Y343F)-SUMO, thereby avoiding the obfuscating effects on DNA binding.

A half-site bound by the Y343F mutant was mixed with one bound by the R191A mutant (Fig 9C), exactly as in cleavage complementation (Fig 9B). As the mutant pair may engage in the interactions responsible for the assembly of the shared active site, the native conditions under which joining occurs are recapitulated. Furthermore, this experimental design makes it possible to test whether the joining activity of a given Y343F mutant protein is differentially affected by whether the partner R191A mutant contains SUMO or not.

Flp(Y343F) and Flp(Y343F)-SUMO gave equal amounts of the hairpin product when the reaction also contained the Flp(R191A)-bound half-site (lanes 1 and 2, Fig 9C) or the half-site bound by Flp(R191A)-SUMO (lanes 3 and 4, Fig 9C). The histogram plots show the relative joining efficiencies for each protein pair normalized against a value of 1.0 for Flp(Y343F) in the presence of Flp(R191A).

Comparable joining activities of native Flp and Flp-SUMO would suggest that enhanced strand joining (or cleavage reversal) by SUMO-modified Flp is probably not a contributing factor in lowering the steady state levels of *FRT* nicks *in vivo*.

### Fewer strand nicks at plasmid *FRT* sites by Flp-SUMO compared to Flp in *siz1Δ siz2 Δ* cells are consistent with lower Flp-SUMO occupancy of these sites

The 2-micron plasmid molecules are organized *in vivo* into nucleosome-beaded mini-chromatin circles [76–78]. There is concern that the *in vitro* behavior of Flp-SUMO towards *FRT* on naked DNA may not be strictly relevant *in vivo*. DNase I sensitivity assays [79] suggest that *FRT* sites in the 2-micron plasmid are relatively nucleosome-free. In order to verify that the *in vitro* differences between Flp and Flp-SUMO are valid *in vivo*, we estimated the amount of strand nicks at *FRT* elicited by a relatively brief but sustained burst of induction of each protein in a cell biological assay. This assay, though indirect, avoids potential problems in quantitating *FRT* nicks by alkaline gel electrophoresis posed by variabilities in the extraction of DNA covalently linked to Flp.

The experimental [Cir^0^] strains, expressing *RAD52*-YFP, contained the p*ADE2*-Flp plasmid, which has two *FRT* sites per molecule (S1 Fig) and a copy number similar to that of the 2-micron plasmid (S2 Fig). Flp or Flp-SUMO was expressed in this strain from the same chromosome locale (*TRP1*; Chromosome IV) under *GAL* promoter control. The small background level of Flp expressed by its native promoter from p*ADE2*-Flp does not interfere with the assay, as it would be swamped out by the galactose-induced levels of Flp or Flp-SUMO. Strand nicks formed at *FRT* were visualized indirectly as Rad52-YFP foci associated with the double strand breaks that such nicks give rise to upon DNA replication [80].

**Fig 10:**
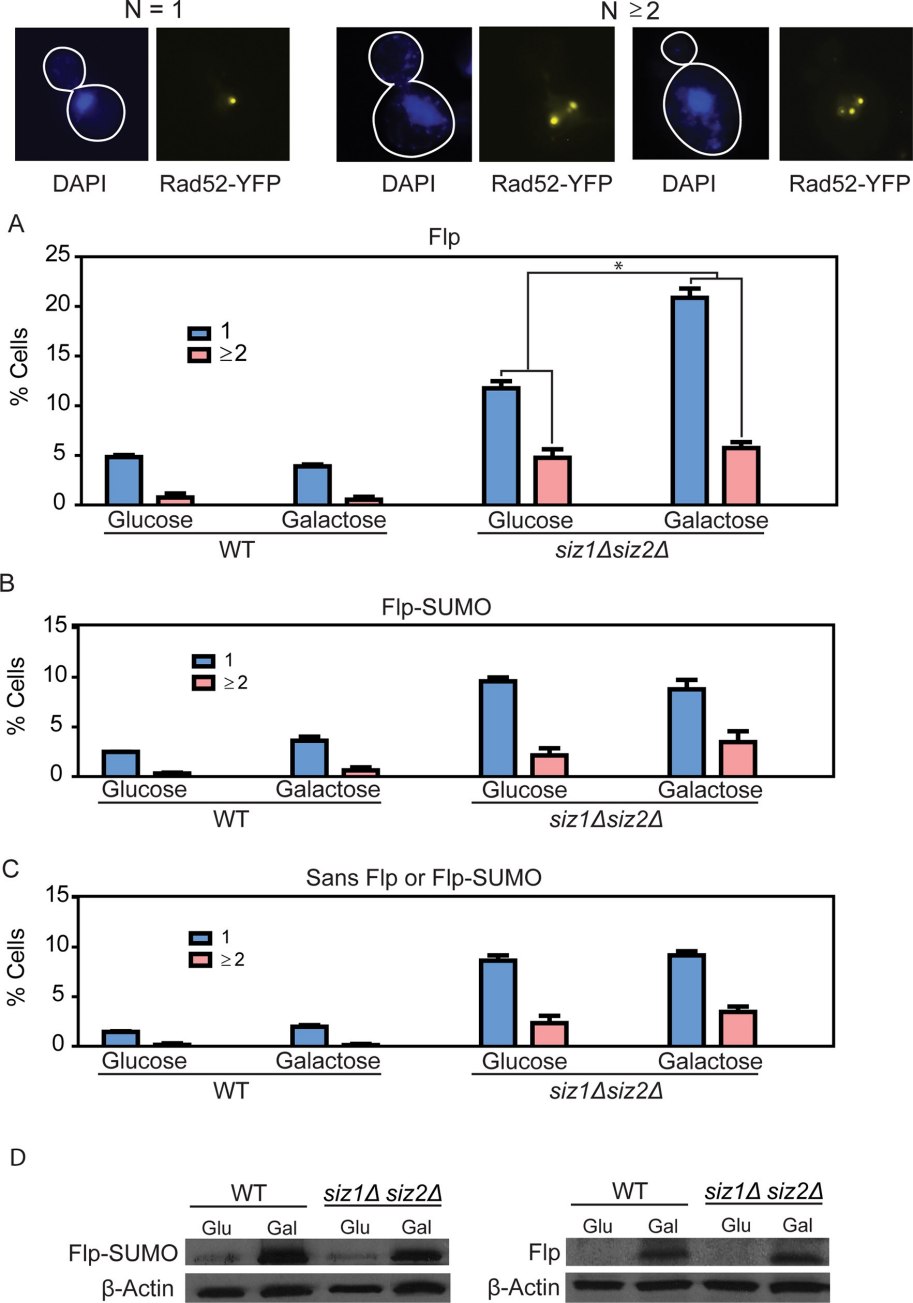
Flp-SUMO mediates fewer strand nicks at plasmid *FRT* sites *in vivo* than Flp. Representative cells containing one, two or more than two Rad52-YFP foci per nucleus (DAPI-stained chromosomes) are shown at the top. Most foci-containing cells displayed a single focus (75–80%). The remainder contained predominantly two foci, and only very few contained three or more. **A**, **B**. Rad52-YFP foci were counted in log-phase cells incubated in medium containing glucose (uninduced) or galactose (induced for Flp or Flp-SUMO expression) for 2 hr at 30°C. Cells were fixed in formaldehyde immediately after the incubation period, and examined by fluorescence microscopy. The plotted data are derived from >250 cells for each pair of histograms representing an experiment. The asterisk indicates p < 0.05. **C**. The assays were done as in **A** and **B**, except that the strains did not contain the *GAL* promoter controlled expression cassettes for Flp or Flp-SUMO. **D**. The levels of the induced proteins were assayed by western blotting, with actin as the control for normalization, as described under Fig 5. Both Flp and Flp-SUMO were detected using an antibody to the HA-tag present in them.

In the wild type strain, the percentage of cells containing Rad52-YFP foci were low (~6% or lower; Fig 10A and 10B) with or without induction of Flp or Flp-SUMO. The foci in uninduced (glucose- grown) cells represent background DNA damage plus any damage at plasmid *FRT* sites due to Flp expressed from p*ADE2*-Flp. The percentage of foci-containing cells was higher in the glucose-grown *siz1Δ* siz2*Δ* strain, (~12 to ~18%) (Fig 10A and 10B). Presumably, this increase signifies general DNA repair deficiency caused by the mutations combined with increased *FRT* strand nicks due to deficient sumoylation of the plasmid-expressed Flp. Induction of Flp by galactose raised the fraction of foci-containing cells from ~18% to ~29% (Fig 10A), signifying a further increase in the frequency of strand nicks at *FRT*. By contrast, there was no such increase upon similar induction of Flp-SUMO (Fig 10B). Our findings are consistent with a previous demonstration of increased Rad52 foci formation due to *siz1Δ siz2Δ* in a [Cir^+^] strain compared to the [Cir^0^] control [33]. In a strain lacking galactose inducible Flp or Flp-SUMO, but containing the p*ADE2*-Flp plasmid, the fractions of Rad52 foci containing cells were not significantly different in glucose- versus galactose-grown *siz1Δ siz2Δ* (Fig 10C). This was also the case for the wild type, as expected.

The striking difference between Flp and Flp-SUMO in foci formation in *siz1Δ siz2Δ* is consistent with the decreased occupancy of *FRT* sites by Flp-SUMO, as suggested by the *in vitro* results. More rapid turnover of Flp-SUMO in *siz1Δ siz2Δ* compared to Flp cannot explain the difference, as these foci were scored immediately following an optimal 2 hr galactose induction of both proteins. In fact, the induced level of Flp-SUMO at this time point was higher than that of Flp in both the wild type and mutant strains (Fig 10D).

## Discussion

**Fig 11:**
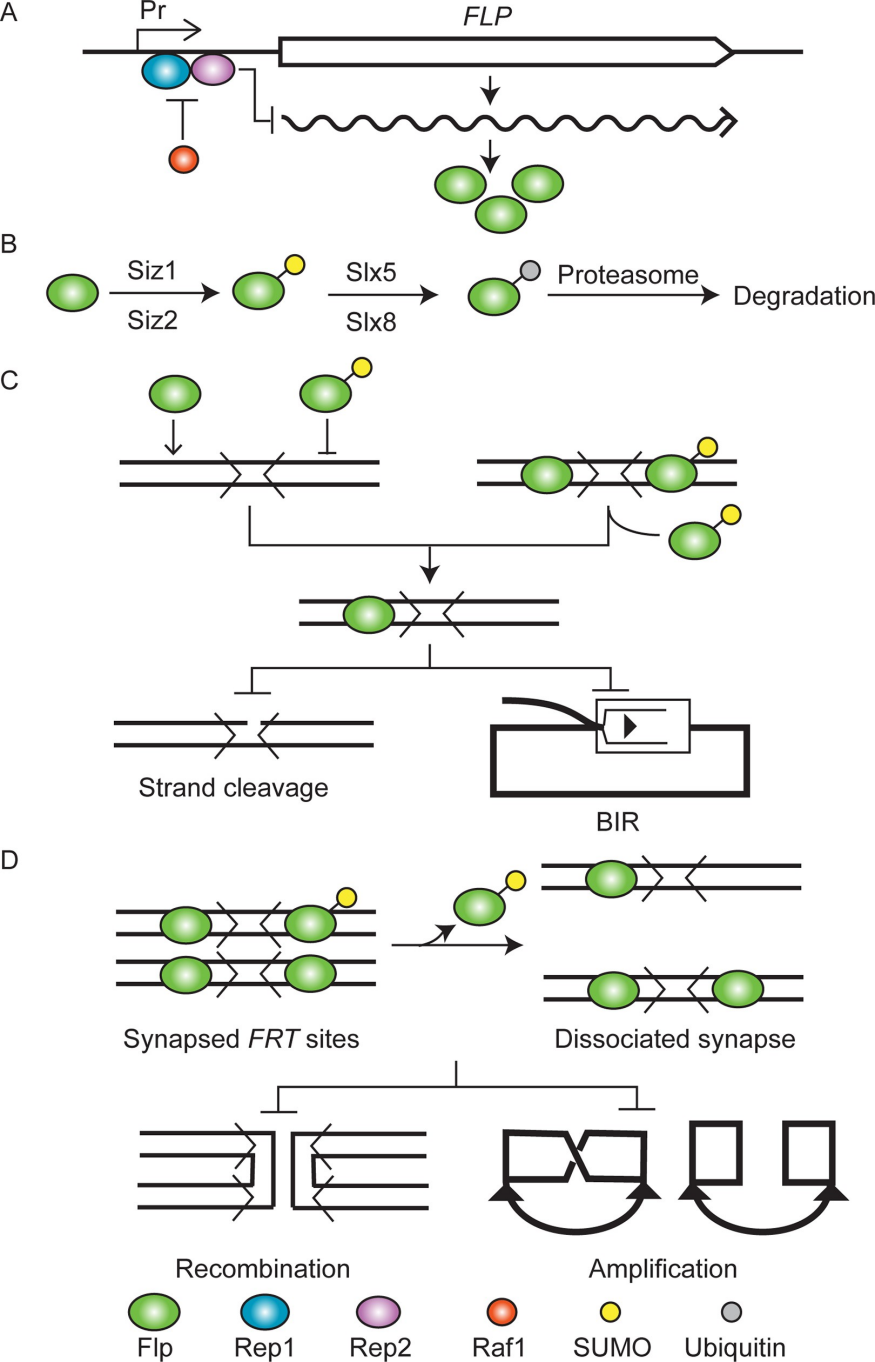
Regulation of Flp in the copy number control of the 2-micron plasmid is tripartite. **A**. The Rep1-Rep2 repressor and its antagonist Raf1 [24,25,27] mediate transcriptional regulation of *FLP*. The *FLP* promoter is indicated by Pr. **B-D**. Sumoylation followed by ubiquitination channels Flp for degradation by the proteasome, thereby controlling its steady state levels [31,33] (this study) (**B**). Decreased association of sumoylated Flp with an *FRT* site, or enhanced dissociation from an occupied site, ameliorates strand cleavage within a site (**C**) or suppresses recombination between partner sites (**D**) (this study). The chances of plasmid overamplification by BIR (Fig 1C) or by replication coupled recombination (Fig 1A and 1B) are thus minimized. The representation of the *FRT* site and its Flp binding elements in **C** and **D** follow the scheme in Figs 7 and 8. In the schematic drawings in **C** and **D**, duplex DNA and single stranded DNA are distinguished by thick and thin lines, respectively, and replication forks are denoted by filled arrowheads.

The *in vivo* and *in vitro* analyses of a Flp-SUMO fusion protein presented here broaden our current understanding of Flp regulation, and provide a comprehensive model for the copy number control of the 2-micron plasmid (Fig 11A–11D). Transcriptional regulation of the *FLP* gene and turnover of post-translationally modified Flp protein balance, at the primary level, the readiness for normal plasmid amplification against the potential risk of aberrant amplification. A secondary layer of fine-tuning to this control is provided through the modulation of target DNA recognition by the modified Flp and of its cooperativity in the context of a dimer required for strand cleavage and a tetramer required for recombination. The model, further elaborated below, has general implications for the mechanisms by which extra-chromosomal selfish DNA elements ensure their evolutionary success by refraining from overburdening their hosts’ metabolic potential.

As the equal segregation efficiency of the 2-micron plasmid is high, the need for plasmid amplification would be quite infrequent at steady state growth conditions. Indeed, analogous to chromosomes, nearly all of the plasmid molecules in a cell population replicate once, and only once, per cell cycle [81]. We do not understand the details of how the negative control of *FLP* expression or of Flp activity is relieved in a cell with a deficit in plasmid copy number caused by missegregation, when this low probability event does occur. The reduced Rep1-Rep2 repressor levels determined by the expression of *REP1* in proportion to plasmid copy number (Rep1 + Rep2 ⇄ [Rep1-Rep2]), together with Raf1-mediated antagonism of the repressor [24,26,27] under this condition, may provide the solution—at least in part. In principle, the increased output of Flp may outpace the post-translational modification system at the level of SUMO conjugation to Flp and/or of SUMO recycling among modified and unmodified Flp molecules. Needless to say that the opposing controls of repression/inactivation versus induction/activation will have to be tightly coordinated in order for Flp to perform its physiological function optimally.

### Flp-SUMO is an authentic substitute for Flp(K375-SUMO)

An important advantage of Flp-SUMO is that it permits direct comparison with unmodified Flp in the DNA binding and cleavage properties of the two purified proteins. We have not utilized *in vitro* SUMO conjugation [82,83] to Flp because of uncertainties in the extent of modification and the potential for conjugation at semi-consensus or non-consensus lysine residues. Structural considerations suggest that the folded SUMO domain linked to Lys-375 or to Ile-423 would be spaced approximately equally from the body of Flp (S4 Table). Furthermore, Flp-SUMO mirrors Flp(Lys-375-SUMO) *in vivo* in that both require Slx5-Slx8 for their normal regulation, while Siz1-Siz2 can be bypassed in the case of Flp-SUMO but not Flp. Equally compelling is the concordance between the reduced *in vitro FRT* binding/cleavage by Flp-SUMO (Figs 7 and 8) and the inferred extents of *in vivo FRT* DNA damage effected by unmodified Flp versus natively modified Flp or Flp-SUMO (Flp >> Flp-SUMO in *siz1Δ siz2Δ* and Flp(K375-SUMO) = Flp-SUMO in wild type) (Fig 10). Extrapolating the interpretations from Flp-SUMO to Flp(K375-SUMO) is therefore justified on multiple grounds.

### Flp-SUMO mimics Flp(K375-SUMO) in blocking BIR-induced hyper-amplification of the 2-micron plasmid

The adverse physiological effects of gross undersumoylation of Flp (*siz1Δ siz2Δ*)—retarded growth, premature cell death and increased plasmid loss—are consistent with BIR-induced hyper-amplification of *FRT*-containing plasmids. These phenotypes are strongly ameliorated by switching from Flp to Flp-SUMO expression. According to prior genetic and biochemical work, BIR is triggered by Flp-mediated strand nicks at *FRT* in conjunction with plasmid replication [31,33]. Mutations in BIR pathways suppress unregulated plasmid amplification [33]. High rates of plasmid loss may occasionally mask the typical BIR phenotypes—as was observed with the hyper-cleaving variant Flp(H305L)—because of the growth advantage of plasmid-cured cells. However, induction of Flp(H305L) leads to cell death when resolution of the branched DNA intermediates of BIR is impaired by *yen1Δ*, *mus81Δ* or *yen1Δ mus81Δ*. Cell killing by Flp(H305L), which correlates with *FRT* copy number, is strongly curbed when these mutants express Flp(H305L)-SUMO instead. The salvaging effects of SUMO fusion to Flp are dependent on the Slx5-Slx8 STUbL, suggesting that SUMO modification of Flp is followed by ubiquitination and proteasome-mediated degradation (Fig 11B). The half-lives of Flp-SUMO in the wild type and mutant strains, and in the presence of the proteasome inhibitor MG-132, are consistent with this scenario. Natively sumoylated Flp has also been shown to be regulated by Slx5-Slx8 [33]. The cumulative results suggest that the mechanisms responsible for SUMO recognition in Flp(K375-SUMO) and downstream processing are shared by Flp-SUMO as well.

### Normal SUMO conjugation and artificial SUMO fusion exert similar biological effects despite the stoichiometric difference in modification

There are precedents for SUMO and ubiquitin fusion proteins satisfying the functional roles of their naturally modified counterparts. Examples in yeast include the recombination protein Rad52 as well as transcriptional regulatory proteins [84–87]. The properties of Flp-SUMO revealed in our studies *vis a vis* the *siz1Δ siz2Δ* and *slx5Δ* or *slx8Δ* host strains are in general conformity with the behavior of other biologically active SUMO fusion proteins.

While sumoylation may signal protein degradation in certain instances, it may act as a safeguard against degradation in others. The opposing functions may be reconciled if the SUMO-acceptor lysine is also the target for ubiquitination by an STUbL [88]. In one case, the addition of ubiquitin may occur in concert with the removal of SUMO by Ulp1. In the other, the pre-existing SUMO may sterically block the action of a ubiquitin ligase. The apparent deregulation of Flp by interfering with either SUMO conjugation (*siz1Δ siz2Δ*) [31,33] or deconjugation (*ulp1*) [34] would fit into the first model. It is not known whether Lys-375 of Flp is the site for both sumoylation and ubiquitination under native conditions. If it is, the functional similarity between Flp and Flp-SUMO suggests that SUMO can activate Slx5-Slx8 in *cis* or in *trans* during ubiquitination.

In spite of the large difference in stoichiometry between SUMO conjugation at Lys-375 (~10%) [31] and SUMO fusion at Ile-423 (100%), the modifications are nearly indistinguishable in their biological roles. Dynamic modification of Lys-375 by deconjugation and reutilization of the SUMO moiety, targeted modification of *FRT*-bound Flp or selective exclusion of modified Flp from *FRT* may overcome the limitations of substoichiometric modification. Deregulation of Flp by *ulp1* [34] would be consistent with the dynamic nature of SUMO conjugated to Lys-375. These general principles—the catalytic nature of the modification, its compartmentalization, and/or its dominance in dimeric or oligomeric protein assemblies—may explain similar biological effects produced by substoichiometric modification of native proteins and stoichiometric modification of the corresponding engineered fusion proteins.

### SUMO conjugation lowers *FRT*-association and *FRT*-cleavage by Flp

As revealed by *in vitro* analyses, Flp-SUMO has weaker affinity for *FRT* than Flp, and is less cooperative in *FRT*-binding. Furthermore, the underrepresentation of *FRT*-bound Flp-SUMO in binding reactions with mixtures of Flp and Flp-SUMO suggests negative cooperativity between the two. As strand cleavage requires the collaboration of two Flp monomers [50,59], the *in vivo* implications of negative cooperativity are significant in the context of a single *FRT* site or a pair of synapsed *FRT* sites. The reduced association or enhanced dissociation of a sumoylated Flp monomer would protect *FRT* from strand cleavage, and the potential initiation of BIR, even if it were stably bound by an unmodified Flp monomer (Fig 11C). Similar dissociation within four recombinase monomers bound to a pair of synapsed *FRT* sites would cause these sites to disengage from each other. Normal plasmid amplification by Flp-mediated reconfiguration of replication forks can thus be regulated as well (Fig 11D).

There are several examples for the modulation of the DNA binding affinity of proteins by SUMO or ubiquitin conjugation, in particular proteins associated with DNA damage repair [65,89]. The modification promotes protein dissociation from DNA in most instances [90–95], although the reverse trend has also been observed [96–98].The properties of Flp-SUMO follow the general theme of fine-tuning DNA-protein interactions through post-translational modification. However, this is the first time that this phenomenon has been demonstrated in the regulation of a site-specific DNA recombinase.

### Self- and host-imposed controls regulate the population of selfish genomes

As an extrachromosomal element, the 2-micron plasmid derives its evolutionary fitness from its high transmission fidelity during cell division, and its ability to restore copy number in case of a glitch during a segregation event [2]. The steady state plasmid copy number of 40–60 molecules in a haploid nucleus is an optimized maximum value. Higher copy numbers reduce the fitness of the host, and indirectly harm the plasmid. Central to copy number maintenance is the regulation of Flp through modulation of gene expression by plasmid-coded proteins (Fig 11A) [24,25,27] and through post-translational modification by the host’s SUMO and ubiquitin conjugation machineries (Fig 11B–11D) [31,33]. The post-translational control is two-pronged. As suggested by previous work [31,33], and confirmed by the present study utilizing Flp-SUMO, proteasome-mediated turnover of Flp, sequentially modified by SUMO and ubiquitin conjugation, lessens the probability of unsealed strand nicks at *FRT* and unwarranted increase in plasmid copy number via BIR (Fig 11B). At another level, as suggested by the *in vitro* and *in vivo* properties of Flp-SUMO, the lower propensity of SUMO-conjugated Flp to stay associated with *FRT* protects it from excessive strand nicks (Fig 11C). By controlling the level and activity of Flp (Fig 11A–11D), SUMO conjugation would also prevent hyper-amplification of the plasmid by *FRT* x *FRT* recombination coupled to plasmid replication (Fig 11D) [21–23].

Thus, safeguards against plasmid overpopulation through moderation of selfishness exercised by the plasmid itself and through preventive measures implemented by the host guarantee the mutual compatibility between a dependent genome and its guardian genome over evolutionary time.

## Supporting information

S1 Fig General characteristics of plasmids expressing Flp or modified forms of Flp are outlined. The plasmid p*ADE2*-Flp, schematically diagrammed at the top, was a derivative of the 2-micron plasmid containing an insertion of the *ADE2* gene at the unique HpaI site within the plasmid genome [41,56]. In addition, the plasmid was engineered to express a version of Flp fused to an HA-His8 epitope at its carboxyl-terminus. Except for these modifications, the rest of the plasmid backbone was the same as that of the native 2-micron circle. The 599 bp inverted repeat sequences, within which the *FRT* sites are embedded, are shown by the horizontal parallel lines. The Flp-*FRT* recombination system and the Rep1-Rep2-*STB* partitioning system were functional in this multi-copy plasmid. In the parent form of p*ADE2*-Flp utilized for the present study, the 2-micron plasmid backbone was in the A-form [42]. Flp-mediated recombination in yeast between the head-to-head *FRT* sites of the plasmid could generate the B-form, and promote A → B and B → A interconversions (see S3 Fig). In p*ADE2*-Flp-SUMO, the *FLP* gene was modified to express a Flp-SUMO hybrid protein in which the mature form of SUMO (amino acids 1–96) was fused to the carboxyl-terminus of Flp via a short peptide linker: Ala-Ser-Gly_4_-Ser. The fusion protein harbored at its carboxyl-terminus the same HA-His8 tag as that fused to Flp. In the p*ADE2*-Flp(H305L) and p*ADE2*-Flp(H305L)-SUMO plasmid derivatives, the active site His-305 in Flp was replaced by leucine. The variants containing the H305L substitution were competent in strand cleavage but were strongly defective in strand joining. The Flp and SUMO moieties are color coded by green and yellow, respectively.
(DOCX)

S2 Fig Plasmid copy numbers are compared in wild type and *siz1Δ siz2Δ* strains. The copy numbers of the native 2-micron plasmid in [Cir^+^] strains and of p*ADE2*-Flp in [Cir^0^] strains (lacking the native plasmid) were estimated by real-time PCR essentially as described previously by Chen et al. [31]. In order that the present and published values are directly comparable, a 65 bp plasmid region and the Y’-subtelomeric element (as reference amplicon) were amplified from total yeast DNA preparations using the primer pairs described by Chen et al. [31]. The sequences of the amplification primers (P1, P2 for plasmid; P3, P4 for chromosome reference) are listed. The relative copy numbers are plotted with a value of 1.0 assigned to 2-micron plasmid in the [Cir^+^] strain. While *siz1Δ siz2Δ* raised the copy number of p*ADE2*-Flp and the native 2-micron plasmid significantly (*, p < 0.05), the relative increase was lower for p*ADE2*-Flp.
(DOCX)

S3 Fig Flp-SUMO is active in mediating recombination between *FRT* sites. **A**. The A-form of the p*ADE2*-Flp-SUMO plasmid introduced into [Cir^0^] strains is shown schematically at the top (left). The B-form resulting from Flp-mediated recombination between the plasmid *FRT* sites is shown to its right. The two forms can be distinguished by the lengths of the PCR products formed with primer 1 (P1) plus primer 2 (P2) or primer 2’ (P2’) using isolated total yeast DNA as the template. Amplified DNA of the expected sizes formed by both primer pairs from p*ADE2*-Flp-SUMO is consistent with the recombinase activity of Flp-SUMO expressed from it. The p*ADE2*-Flp plasmid expressing wild type Flp served as a positive control for recombination. The larger product sizes from p*ADE2*-Flp-SUMO are consistent with the increased length of the hybrid *FLP*-SUMO locus. **B**. Since p*ADE2*-Flp-SUMO contains a 599 bp long inverted repeat sequence, each with an embedded *FRT* site, a strand break within one *FRT* may be repaired by using the second intact copy of the repeat as template. Resolution of a potential Holliday junction intermediate of repair in the crossover mode would produce the B-form plasmid from the parental A-form. In order to eliminate the possibility of repair-mediated A- to B-form conversion, a second recombination assay was performed in a [Cir^0^] strain containing a substrate plasmid in which two minimal *FRT* sites (each 34 bp long) bordered *TRP1* in the head-to-tail orientation. The strain was engineered to express Flp-SUMO from a chromosomal locus under the control of the *GAL* promoter. Aliquots from overnight cultures grown under non-inducing (glucose) and inducing (galactose) conditions were plated on medium lacking uracil, and 3-day old colonies were replica-plated and grown on medium without tryptophan. The loss of the *TRP1* marker in galactose-grown cells verifies *FRT* x *FRT* recombination mediated by Flp-SUMO. Expression of Flp-SUMO(Y343F) did not yield *TRP1* deletion.
(DOCX)

S4 Fig Native Flp and Flp epitope-tagged at its carboxyl-terminus are equally active in recombination *in vitro*. Native Flp and Flp-HA-His8 were purified to near homogeneity using *E*. *coli* expression systems [43–45]. The excision reaction (schematically illustrated at the top) was performed as outlined in the legend to (Fig 6A and 6B). The EcoRV plus NdeI digestion products formed from the substrate plasmid and the excision circles are indicated by ‘S’ and ‘P’, respectively.
(DOCX)

S1 Table Yeast strains and their relevant features. The genotypes of the yeast strains used in the present study along with the figures/table depicting the experimental results obtained with them are listed.
(DOCX)

S2 Table Plasmids. Yeast and bacterial plasmids utilized in this work and their relevant features are summarized. The figures/table containing experimental data obtained using these plasmids are indicated. The Flp and Flp-SUMO proteins and their H305L variants expressed from the yeast plasmids contained the HA-His8 tag at their carboxyl-terminus. The pCM66 and pCM331 integrative plasmids were inserted into the *leu2* locus of the experimental yeast strains. The pBAD33-derived plasmids were used for the expression and purification of Flp with or without the carboxyl-terminal HA-His8 tag as well as Flp-SUMO containing this tag. The pBAD24-derived plasmid served as the substrate for *in vitro* recombination assays.
(DOCX)

S3 Table Nibbled colonies and mini-colonies induced by p*ADE2*-Flp or its derivatives are quantified in wild type and mutant strains. Representative colony types observed are shown at the top: **A**. smooth white (normal) colonies harboring an *ADE2* containing reporter plasmid; **B**. plasmid containing nibbled and growth-stunted (mini) colonies; **C**. a smooth red colony formed by a plasmid-free cell (left) and a colony with a smooth red (plasmid-free) sector and a nibbled white (plasmid containing) sector (right). The colony morphologies of the wild type [Cir^0^], *siz1Δ siz2Δ* [Cir^0^], *slx5Δ* [Cir^0^] and *slx8Δ* [Cir^0^] strains harboring the indicated plasmids were quantitated from the assays depicted in Figs 2 and 4 and similar assays. The listed values represent the combined averages from cell populations plated on YEPD medium at n = 0 (overnight culture in selective medium lacking adenine) and at n = 10 (after n = 0 cells were grown non-selectively in liquid medium for 10 generations). The liquid cultures were incubated at 30°C. The plate cultures derived from n = 0 and n = 10 liquid cultures were grown at 26°C (5 days) before screening colony morphology (a total of > 800 colonies from n = 0 and n = 10 plates for each assay). As explained in the legend to Fig 2, only white colonies and the white patches within sectored colonies were scored for the presence of nibbled edges. Mini-colonies were so designated when they were conspicuously smaller than the average sized colonies on 5-day incubated plates. Completely red colonies, indicating plasmid loss before plating, were omitted from consideration. WT = wild type strain.
(DOCX)

S4 Table Relative dispositions of Lys-375 and the carboxyl-terminus of Flp with respect to the catalytic hexad residues of the active site are displayed. The distances between α-carbon atoms assembled in panel **A** are for a Flp monomer (bound adjacent to a cleaved scissile phosphate). In this structure (PDB, 1M6X), the polypeptide chain ends with Arg-422; Ile-423 is not visible [70]. Similar distances arranged in panel **B** are for a Flp monomer (bound adjacent to an uncleaved scissile phosphate). The carboxyl-terminal I-423 is visible in this structure (PDB, 1FLO) [59].
(DOCX)

## References

[1] Broach JR, Volkert FC (1991) Circular DNA Plasmids of Yeasts: The Molecular Biology of the Yeast *Saccharomyces* Genome Dynamics, Protein Synthesis and Energetics. Broach JR, Pringle JR, Jones EW, editors. Cold Spring harbor, New York: Cold Spring Harbor Laboratory Press. 297–331 p.

[2] Liu Y-T, Ma C-H, Kachroo AH, Rowley PA, Chang KM, et al (2015) The partitioning and copy number control systems of the selfish yeast plasmid: an optimized design for stable persistence in host cells: Plasmids: Biology and Impact in Biotechnology and Discovery. Tolmasky M, Alonso JC, editors. Washington DC: ASM Press. 325–348 p.

[3] Rizvi SMA, Prajapati HK, Ghosh SK (2018) The 2 micron plasmid: a selfish genetic element with an optimized survival strategy within Saccharomyces cerevisiae. Curr Genet 64: 25–42. 10.1007/s00294-017-0719-2 28597305

[4] Sau S, Ghosh SK, Liu YT, Ma CH, Jayaram M (2019) Hitchhiking on chromosomes: A persistence strategy shared by diverse selfish DNA elements. Plasmid 102: 19–28. 10.1016/j.plasmid.2019.01.004 30726706

[5] Jayaram M, Li YY, Broach JR (1983) The yeast plasmid 2 micron circle encodes components required for its high copy propagation. Cell 34: 95–104. 6883512 10.1016/0092-8674(83)90139-3

[6] Kikuchi Y (1983) Yeast plasmid requires a cis-acting locus and two plasmid proteins for its stable maintenance. Cell 35: 487–493. 6317192 10.1016/0092-8674(83)90182-4

[7] McQuaid ME, Polvi EJ, Dobson MJ (2018) DNA sequence elements required for partitioning competence of the Saccharomyces cerevisiae 2-micron plasmid STB locus. Nucleic Acids Res.47: 716–728. 10.1093/nar/gky1150 PMC6344848 30445476

[8] Ghosh SK, Hajra S, Jayaram M (2007) Faithful segregation of the multicopy yeast plasmid through cohesin-mediated recognition of sisters. Proc Natl Acad Sci USA 104: 13034–13039. 10.1073/pnas.0702996104 17670945 PMC1941829

[9] Liu YT, Chang KM, Ma CH, Jayaram M (2016) Replication-dependent and independent mechanisms for the chromosome-coupled persistence of a selfish genome. Nucleic Acids Res 44: 8302–8323. 10.1093/nar/gkw694 27492289 PMC5041486

[10] Liu YT, Ma CH, Jayaram M (2013) Co-segregation of yeast plasmid sisters under monopolin-directed mitosis suggests association of plasmid sisters with sister chromatids. Nucleic Acids Res 41: 4144–4158. 10.1093/nar/gkt096 23423352 PMC3627588

[11] Sau S, Conrad MN, Lee CY, Kaback DB, Dresser ME, et al (2014) A selfish DNA element engages a meiosis-specific motor and telomeres for germ-line propagation. J Cell Biol 205: 643–661. 10.1083/jcb.201312002 24914236 PMC4050733

[12] Barbera AJ, Chodaparambil JV, Kelley-Clarke B, Joukov V, Walter JC, et al (2006) The nucleosomal surface as a docking station for Kaposi's sarcoma herpesvirus LANA. Science 311: 856–861. 10.1126/science.1120541 16469929

[13] Botchan M (2004) Hitchhiking without covalent integration. Cell 117: 280–281. 15109488 10.1016/s0092-8674(04)00410-6

[14] Frappier L (2004) Viral plasmids in mammalian cells: Plasmid Biology. Phillips G, Funnell BE, editors. Washington DC: ASM Press. 325–340 p.

[15] Ilves I, Kivi S, Ustav M (1999) Long-term episomal maintenance of bovine papillomavirus type 1 plasmids is determined by attachment to host chromosomes, which is mediated by the viral E2 protein and its binding sites. J Virol 73: 4404–4412. 10196338 10.1128/jvi.73.5.4404-4412.1999 PMC104221

[16] Kanda T, Kamiya M, Maruo S, Iwakiri D, Takada K (2007) Symmetrical localization of extrachromosomally replicating viral genomes on sister chromatids. J Cell Sci 120: 1529–1539. 10.1242/jcs.03434 17405814

[17] McBride AA (2008) Replication and partitioning of papillomavirus genomes. Adv Virus Res 72: 155–205. 10.1016/S0065-3527(08)00404-1 19081491 PMC3151303

[18] Nanbo A, Sugden A, Sugden B (2007) The coupling of synthesis and partitioning of EBV's plasmid replicon is revealed in live cells. EMBO J 26: 4252–4262. 10.1038/sj.emboj.7601853 17853891 PMC2000340

[19] You J, Croyle JL, Nishimura A, Ozato K, Howley PM (2004) Interaction of the bovine papillomavirus E2 protein with Brd4 tethers the viral DNA to host mitotic chromosomes. Cell 117: 349–360. 15109495 10.1016/s0092-8674(04)00402-7

[20] You J, Srinivasan V, Denis GV, Harrington WJ Jr, Ballestas ME, et al (2006) Kaposi's sarcoma-associated herpesvirus latency-associated nuclear antigen interacts with bromodomain protein Brd4 on host mitotic chromosomes. J Virol 80: 8909–8919. 10.1128/JVI.00502-06 16940503 PMC1563901

[21] Futcher AB (1986) Copy number amplification of the 2 micron circle plasmid of Saccharomyces cerevisiae. J Theor Biol 119: 197–204. 3525993 10.1016/s0022-5193(86)80074-1

[22] Volkert FC, Broach JR (1986) Site-specific recombination promotes plasmid amplification in yeast. Cell 46: 541–550. 3524855 10.1016/0092-8674(86)90879-2

[23] Petes TD, Williamson DH (1994) A novel structural form of the 2 micron plasmid of the yeast Saccharomyces cerevisiae. Yeast 10: 1341–1345. 10.1002/yea.320101011 7900423

[24] Murray JA, Scarpa M, Rossi N, Cesareni G (1987) Antagonistic controls regulate copy number of the yeast 2 micron plasmid. EMBO J 6: 4205–4212. 2832156 10.1002/j.1460-2075.1987.tb02768.x PMC553905

[25] Reynolds AE, Murray AW, Szostak JW (1987) Roles of the 2 micron gene products in stable maintenance of the 2 micron plasmid of Saccharomyces cerevisiae. Mol Cell Biol 7: 3566–3573. 10.1128/mcb.7.10.3566 3316982 PMC368010

[26] Rizvi SMA, Prajapati HK, Nag P, Ghosh SK (2017) The 2-micron plasmid encoded protein Raf1 regulates both stability and copy number of the plasmid by blocking the formation of the Rep1-Rep2 repressor complex. Nucleic Acids Res 45: 7167–7179. 10.1093/nar/gkx316 28472368 PMC5499539

[27] Som T, Armstrong KAF, Volkert C, Broach JR (1988) Autoregulation of 2 micron circle gene expression provides a model for maintenance of stable plasmid copy levels. Cell 52: 27–37. 2449970 10.1016/0092-8674(88)90528-4

[28] Jayaram M, Yang XM, Mehta S, Voziyanov Y, Velmurugan S (2004) The 2 micron plasmid of *Saccharomyces cerevisiae*: Plasmid Biology. Phillips G, Funnell BE, editors. Washington DC: ASM Press. 303–324 p.

[29] Velmurugan S, Mehta S, Jayaram M (2003) Selfishness in moderation: evolutionary success of the yeast plasmid. Curr Top Dev Biol 56: 1–24. 14584724 10.1016/s0070-2153(03)01005-6

[30] McQuaid ME, Pinder JB, Arumuggam N, Lacoste JSC, Chew JSK, et al (2017) The yeast 2-micron plasmid Raf protein contributes to plasmid inheritance by stabilizing the Rep1 and Rep2 partitioning proteins. Nucleic Acids Res 45: 10518–10533. 10.1093/nar/gkx703 29048592 PMC5737570

[31] Chen XL, Reindle A, Johnson ES (2005) Misregulation of 2 micron circle copy number in a SUMO pathway mutant. Mol Cell Biol 25: 4311–4320. 10.1128/MCB.25.10.4311-4320.2005 15870299 PMC1087719

[32] Pinder JB, McQuaid ME, Dobson MJ (2013) Deficient sumoylation of yeast 2-micron plasmid proteins Rep1 and Rep2 associated with their loss from the plasmid-partitioning locus and impaired plasmid inheritance. PLoS One 8: e60384 10.1371/journal.pone.0060384 23555963 PMC3610928

[33] Xiong L, Chen XL, Silver HR, Ahmed NT, Johnson ES (2009) Deficient SUMO attachment to Flp recombinase leads to homologous recombination-dependent hyperamplification of the yeast 2 micron circle plasmid. Mol Biol Cell 20: 1241–1251. 10.1091/mbc.E08-06-0659 19109426 PMC2642740

[34] Dobson MJ, Pickett AJ, Velmurugan S, Pinder JB, Barrett LA, et al (2005) The 2 micron plasmid causes cell death in Saccharomyces cerevisiae with a mutation in Ulp1 protease. Mol Cell Biol 25: 4299–4310. 10.1128/MCB.25.10.4299-4310.2005 15870298 PMC1087720

[35] Holm C (1982) Clonal lethality caused by the yeast plasmid 2 micron DNA. Cell 29: 585–594. 7116450 10.1016/0092-8674(82)90174-x

[36] Burgess RC, Rahman S, Lisby M, Rothstein R, Zhao X (2007) The Slx5-Slx8 complex affects sumoylation of DNA repair proteins and negatively regulates recombination. Mol Cell Biol 27: 6153–6162. 10.1128/MCB.00787-07 17591698 PMC1952148

[37] Ii T, Mullen JR, Slagle CE, Brill SJ (2007) Stimulation of in vitro sumoylation by Slx5-Slx8: evidence for a functional interaction with the SUMO pathway. DNA Repair (Amst) 6: 1679–1691. 10.1016/j.dnarep.2007.06.004 17669696 PMC2100399

[38] Zhao X, Wu CY, Blobel G (2004) Mlp-dependent anchorage and stabilization of a desumoylating enzyme is required to prevent clonal lethality. J Cell Biol 167: 605–611. 10.1083/jcb.200405168 15557117 PMC2172573

[39] McEachern MJ, Haber JE (2006) Break-induced replication and recombinational telomere elongation in yeast. Annu Rev Biochem 75: 111–135. 10.1146/annurev.biochem.74.082803.133234 16756487

[40] Tomaska L, Nosek J, Kramara J, Griffith JD (2009) Telomeric circles: universal players in telomere maintenance? Nat Struct Mol Biol 16: 1010–1015. 10.1038/nsmb.1660 19809492 PMC4041010

[41] Ma CH, Cui H, Hajra S, Rowley PA, Fekete C, et al (2013) Temporal sequence and cell cycle cues in the assembly of host factors at the yeast 2 micron plasmid partitioning locus. Nucleic Acids Res 41: 2340–2353. 10.1093/nar/gks1338 23275556 PMC3575823

[42] Hartley JL, Donelson JE (1980) Nucleotide sequence of the yeast plasmid. Nature 286: 860–865. 6251374 10.1038/286860a0

[43] Lee J, Whang I, Jayaram M (1996) Assembly and orientation of Flp recombinase active sites on two-, three- and four-armed DNA substrates: implications for a recombination mechanism. J Mol Biol 257: 532–549. 10.1006/jmbi.1996.0183 8648622

[44] Meyer-Leon L, Gates CA, Attwood JM, Wood EA, Cox MM (1987) Purification of the FLP site-specific recombinase by affinity chromatography and re-examination of basic properties of the system. Nucleic Acids Res 15: 6469–6488. 10.1093/nar/15.16.6469 3306602 PMC306117

[45] Parsons RL, Evans BR, Zheng L, Jayaram M (1990) Functional analysis of Arg-308 mutants of Flp recombinase. Possible role of Arg-308 in coupling substrate binding to catalysis. J Biol Chem 265: 4527–4533. 2407737

[46] Murray JA, Cesareni G (1986) Functional analysis of the yeast plasmid partition locus STB. EMBO J 5: 3391–3399. 16453734 10.1002/j.1460-2075.1986.tb04655.x PMC1167338

[47] Livak KJ, Schmittgen TD (2001) Analysis of relative gene expression data using real-time quantitative PCR and the 2^-*ΔΔC*(T)^ method. Methods 25: 402–408. 10.1006/meth.2001.1262 11846609

[48] Liu C, Apodaca J, Davis LE, Rao H (2007) Proteasome inhibition in wild-type yeast Saccharomyces cerevisiae cells. Biotechniques 42: 158, 160, 162. 10.2144/000112389 17373478

[49] Prasad PV, Young LJ, Jayaram M (1987) Mutations in the 2-micron circle site-specific recombinase that abolish recombination without affecting substrate recognition. Proc Natl Acad Sci USA 84: 2189–2193. 10.1073/pnas.84.8.2189 3104911 PMC304614

[50] Chen JW, Lee J, Jayaram M (1992) DNA cleavage in trans by the active site tyrosine during Flp recombination: switching protein partners before exchanging strands. Cell 69: 647–658. 1586945 10.1016/0092-8674(92)90228-5

[51] Ma CH, Rowley PA, Macieszak A, Guga P, Jayaram M (2009) Active site electrostatics protect genome integrity by blocking abortive hydrolysis during DNA recombination. EMBO J 28: 1745–1756. 10.1038/emboj.2009.131 19440204 PMC2699359

[52] Holm C (1982) Sensitivity to the yeast plasmid 2 micron DNA is conferred by the nuclear allele nibl. Mol Cell Biol 2: 985–992. 10.1128/mcb.2.8.985 6752695 PMC369886

[53] Mayle R, Campbell IM, Beck CR, Yu Y, Wilson M, et al (2015) DNA Repair. Mus81 and converging forks limit the mutagenicity of replication fork breakage. Science 349: 742–747. 10.1126/science.aaa8391 26273056 PMC4782627

[54] Nielsen I, Bentsen IB, Lisby M, Hansen S, Mundbjerg K, et al (2009) A Flp-nick system to study repair of a single protein-bound nick in vivo. Nat Meth 6: 753–757. 10.1038/nmeth.1372 19749762

[55] Parsons RL, Prasad PV, Harshey RM, Jayaram M (1988) Step-arrest mutants of FLP recombinase: implications for the catalytic mechanism of DNA recombination. Mol Cell Biol 8: 3303–3310. 10.1128/mcb.8.8.3303 2974924 PMC363564

[56] Tsalik EL, Gartenberg MR (1998) Curing Saccharomyces cerevisiae of the 2 micron plasmid by targeted DNA damage. Yeast 14: 847–852. 10.1002/(SICI)1097-0061(19980630)14:9<847::AID-YEA285>3.0.CO;2-9 9818722

[57] Anand RP, Lovett ST, Haber JE (2013) Break-induced DNA replication. Cold Spring Harb Perspect Biol 5: a010397 10.1101/cshperspect.a010397 23881940 PMC3839615

[58] Malkova A, Ira G (2013) Break-induced replication: functions and molecular mechanism. Curr Opin Genet Dev 23: 271–279. 10.1016/j.gde.2013.05.007 23790415 PMC3915057

[59] Chen Y, Narendra U, Iype LE, Cox MM, Rice PA (2000) Crystal structure of a Flp recombinase-Holliday junction complex: assembly of an active oligomer by helix swapping. Mol Cell 6: 885–897. 11090626

[60] Lee J, Jayaram M (1997) A tetramer of the Flp recombinase silences the trimers within it during resolution of a Holliday junction substrate. Genes Dev 11: 2438–2447. 10.1101/gad.11.18.2438 9308970 PMC316519

[61] Lee J, Tonozuka T, Jayaram M (1997) Mechanism of active site exclusion in a site-specific recombinase: role of the DNA substrate in conferring half-of-the-sites activity. Genes Dev 11: 3061–3071. 10.1101/gad.11.22.3061 9367987 PMC316700

[62] Abed M, Bitman-Lotan E, Orian A (2018) The biology of SUMO-targeted ubiquitin ligases in Drosophila development, immunity, and cancer. J Dev Biol 6: pii, E2. 10.3390/jdb6010002 PMC5875560 29615551

[63] Nie M, Boddy MN (2016) Cooperativity of the SUMO and ubiquitin pathways in genome stability. Biomolecules 6: 14 10.3390/biom6010014 26927199 PMC4808808

[64] Perry JJ, Tainer JA, Boddy MN (2008) A SIM-ultaneous role for SUMO and ubiquitin. Trends Biochem Sci 33: 201–208. 10.1016/j.tibs.2008.02.001 18403209

[65] Sarangi P, Zhao X (2015) SUMO-mediated regulation of DNA damage repair and responses. Trends Biochem Sci 40: 233–242. 10.1016/j.tibs.2015.02.006 25778614 PMC4380773

[66] Sriramachandran AM, Dohmen RJ (2014) SUMO-targeted ubiquitin ligases. Biochim Biophys Acta 1843: 75–85. 10.1016/j.bbamcr.2013.08.022 24018209

[67] Wang Z, Prelich G (2009) Quality control of a transcriptional regulator by SUMO-targeted degradation. Mol Cell Biol 29: 1694–1706. 10.1128/MCB.01470-08 19139279 PMC2655623

[68] Xie Y, Rubenstein EM, Matt T, Hochstrasser M (2010) SUMO-independent in vivo activity of a SUMO-targeted ubiquitin ligase toward a short-lived transcription factor. Genes Dev 24: 893–903. 10.1101/gad.1906510 20388728 PMC2861189

[69] Horigome C, Bustard DE, Marcomini I, Delgoshaie N, Tsai-Pflugfelder M, et al (2016) PolySUMOylation by Siz2 and Mms21 triggers relocation of DNA breaks to nuclear pores through the Slx5/Slx8 STUbL. Genes Dev 30: 931–945. 10.1101/gad.277665.116 27056668 PMC4840299

[70] Conway AB, Chen Y, Rice PA (2003) Structural plasticity of the Flp-Holliday junction complex. J Mol Biol 326: 425–434. 12559911 10.1016/s0022-2836(02)01370-0

[71] Pan G, Luetke K, Sadowski PD (1993) Mechanism of cleavage and ligation by FLP recombinase: classification of mutations in FLP protein by in vitro complementation analysis. Mol Cell Biol 13: 3167–3175. 10.1128/mcb.13.6.3167 8497247 PMC359755

[72] Pan G, Luetke K, Juby CD, Brousseau R, Sadowski P (1993) Ligation of synthetic activated DNA substrates by site-specific recombinases and topoisomerase I. J Biol Chem 268: 3683–3689. 8381436

[73] Qian XH, Inman RB, Cox MM (1992) Reactions between half- and full-FLP recombination target sites. A model system for analyzing early steps in FLP protein-mediated site-specific recombination. J Biol Chem 267: 7794–7805. 1560013

[74] Serre MC, Evans BR, Araki H, Oshima Y, Jayaram M (1992) Half-site recombinations mediated by yeast site-specific recombinases Flp and R. J Mol Biol 225: 621–642. 1602474 10.1016/0022-2836(92)90390-6

[75] Lee J, Jayaram M (1995) Functional roles of individual recombinase monomers in strand breakage and strand union during site-specific DNA recombination. J Biol Chem 270: 23203–23211. 10.1074/jbc.270.39.23203 7559468

[76] Livingston DM, Hahne S (1979) Isolation of a condensed, intracellular form of the 2-micron DNA plasmid of Saccharomyces cerevisiae. Proc Natl Acad Sci USA 76: 3727–3731. 10.1073/pnas.76.8.3727 386345 PMC383906

[77] Nelson RG, Fangman WL (1979) Nucleosome organization of the yeast 2-micron DNA plasmid: a eukaryotic minichromosome. Proc Natl Acad Sci USA 76: 6515–6519. 10.1073/pnas.76.12.6515 392520 PMC411896

[78] Seligy VL, Thomas DY, Miki BL (1980) Saccharomyces cerevisiae plasmid, Scp or 2 micron: intracellular distribution, stability and nucleosomal-like packaging. Nucleic Acids Res 8: 3371–3391. 10.1093/nar/8.15.3371 6255414 PMC324158

[79] Fagrelius TJ, Livingston DM (1984) Location of DNAase I sensitive cleavage sites in the yeast 2 micron plasmid DNA chromosome. J Mol Biol 173: 1–13. 6230456 10.1016/0022-2836(84)90400-5

[80] Lisby M, Rothstein R, Mortensen UH (2001) Rad52 forms DNA repair and recombination centers during S phase. Proc Natl Acad Sci USA 98: 8276–8282. 10.1073/pnas.121006298 11459964 PMC37432

[81] Zakian VA, Brewer BJ, Fangman WL (1979) Replication of each copy of the yeast 2 micron DNA plasmid occurs during the S phase. Cell 17: 923–934. 385147 10.1016/0092-8674(79)90332-5

[82] Kolesar P, Altmannova V, Silva S, Lisby M, Krejci L (2016) Pro-recombination Role of Srs2 Protein Requires SUMO (Small Ubiquitin-like Modifier) but Is Independent of PCNA (Proliferating Cell Nuclear Antigen) Interaction. J Biol Chem 291: 7594–7607. 10.1074/jbc.M115.685891 26861880 PMC4817187

[83] Kolesar P, Sarangi P, Altmannova V, Zhao X, Krejci L (2012) Dual roles of the SUMO-interacting motif in the regulation of Srs2 sumoylation. Nucleic Acids Res 40: 7831–7843. 10.1093/nar/gks484 22705796 PMC3439891

[84] Esta A, Ma E, Dupaigne P, Maloisel L, Guerois R, et al (2013) Rad52 sumoylation prevents the toxicity of unproductive Rad51 filaments independently of the anti-recombinase Srs2. PLoS Genet 9: e1003833 10.1371/journal.pgen.1003833 24130504 PMC3794917

[85] Holmstrom S, Van Antwerp ME, Iniguez-Lluhi JA (2003) Direct and distinguishable inhibitory roles for SUMO isoforms in the control of transcriptional synergy. Proc Natl Acad Sci USA 100: 15758–15763. 10.1073/pnas.2136933100 14663148 PMC307641

[86] Ross S, Best JL, Zon LI, Gill G (2002) SUMO-1 modification represses Sp3 transcriptional activation and modulates its subnuclear localization. Mol Cell 10: 831–842. 12419227 10.1016/s1097-2765(02)00682-2

[87] Salghetti SE, Caudy AA, Chenoweth JG, Tansey WP (2001) Regulation of transcriptional activation domain function by ubiquitin. Science 293: 1651–1653. 10.1126/science.1062079 11463878

[88] Prudden J, Pebernard S, Raffa G, Slavin DA, Perry JJ, et al (2007) SUMO-targeted ubiquitin ligases in genome stability. EMBO J 26: 4089–4101. 10.1038/sj.emboj.7601838 17762865 PMC2230673

[89] Ulrich HD (2014) Two-way communications between ubiquitin-like modifiers and DNA. Nat Struct Mol Biol 21: 317–324. 10.1038/nsmb.2805 24699080

[90] Hardeland U, Steinacher R, Jiricny J, Schar P (2002) Modification of the human thymine-DNA glycosylase by ubiquitin-like proteins facilitates enzymatic turnover. EMBO J 21: 1456–1464. 10.1093/emboj/21.6.1456 11889051 PMC125358

[91] Altmannova V, Eckert-Boulet N, Arneric M, Kolesar P, Chaloupkova R, et al (2010) Rad52 SUMOylation affects the efficiency of the DNA repair. Nucleic Acids Res 38: 4708–4721. 10.1093/nar/gkq195 20371517 PMC2919706

[92] Sarangi P, Bartosova Z, Altmannova V, Holland C, Chavdarova M, et al (2014) Sumoylation of the Rad1 nuclease promotes DNA repair and regulates its DNA association. Nucleic Acids Res 42: 6393–6404. 10.1093/nar/gku300 24753409 PMC4041466

[93] Vigasova D, Sarangi P, Kolesar P, Vlasakova D, Slezakova Z, et al (2013) Lif1 SUMOylation and its role in non-homologous end-joining. Nucleic Acids Res 41: 5341–5353. 10.1093/nar/gkt236 23571759 PMC3664818

[94] Sridharan V, Park H, Ryu H, Azuma Y (2015) SUMOylation regulates polo-like kinase 1-interacting checkpoint helicase (PICH) during mitosis. J Biol Chem 290: 3269–3276. 10.1074/jbc.C114.601906 25564610 PMC4319000

[95] Wu SY, Chiang CM (2009) Crosstalk between sumoylation and acetylation regulates p53-dependent chromatin transcription and DNA binding. EMBO J 28: 1246–1259. 10.1038/emboj.2009.83 19339993 PMC2683057

[96] Hang LE, Lopez CR, Liu X, Williams JM, Chung I, et al (2014) Regulation of Ku-DNA association by Yku70 C-terminal tail and SUMO modification. J Biol Chem 289: 10308–10317. 10.1074/jbc.M113.526178 24567323 PMC4036155

[97] Takahashi Y, Yong-Gonzalez V, Kikuchi Y, Strunnikov A (2006) SIZ1/SIZ2 control of chromosome transmission fidelity is mediated by the sumoylation of topoisomerase II. Genetics 172: 783–794. 10.1534/genetics.105.047167 16204216 PMC1456244

[98] Sato K, Toda K, Ishiai M, Takata M, Kurumizaka H (2012) DNA robustly stimulates FANCD2 monoubiquitylation in the complex with FANCI. Nucleic Acids Res 40: 4553–4561. 10.1093/nar/gks053 22287633 PMC3378891

