# Supplementary material for: A Flp-SUMO hybrid recombinase reveals multi-layered copy number control of a selfish DNA element through post-translational modification
Source: PLoS Genet. 2019 Jun 26;15(6):e1008193. doi: 10.1371/journal.pgen.1008193 (PMC6594588; doi:10.1371/journal.pgen.1008193)
Supplement: S1 Fig — The plasmid pADE2-Flp, schematically diagrammed at the top, was a derivative of the 2-micron plasmid containing an insertion of the ADE2 gene at the unique HpaI site within the plasmid genome [41,56]. In addition, the plasmid was engineered to express a version of Flp fused to an HA-His8 epitope at its carboxyl-terminus. Except for these modifications, the rest of the plasmid backbone was the same as that of the native 2-micron circle. The 599 bp inverted repeat sequences, within which the FRT sites are embedded, are shown by the horizontal parallel lines. The Flp-FRT recombination system and the Rep1-Rep2-STB partitioning system were functional in this multi-copy plasmid. In the parent form of pADE2-Flp utilized for the present study, the 2-micron plasmid backbone was in the A-form [42]. Flp-mediated recombination in yeast between the head-to-head FRT sites of the plasmid could generate the B-form, and promote A → B and B → A interconversions (see S3 Fig). In pADE2-Flp-SUMO, the FLP gene was modified to express a Flp-SUMO hybrid protein in which the mature form of SUMO (amino acids 1–96) was fused to the carboxyl-terminus of Flp via a short peptide linker: Ala-Ser-Gly4-Ser. The fusion protein harbored at its carboxyl-terminus the same HA-His8 tag as that fused to Flp. In the pADE2-Flp(H305L) and pADE2-Flp(H305L)-SUMO plasmid derivatives, the active site His-305 in Flp was replaced by leucine. The variants containing the H305L substitution were competent in strand cleavage but were strongly defective in strand joining. The Flp and SUMO moieties are color coded by green and yellow, respectively. (DOCX) [file pgen.1008193.s001.docx]

**
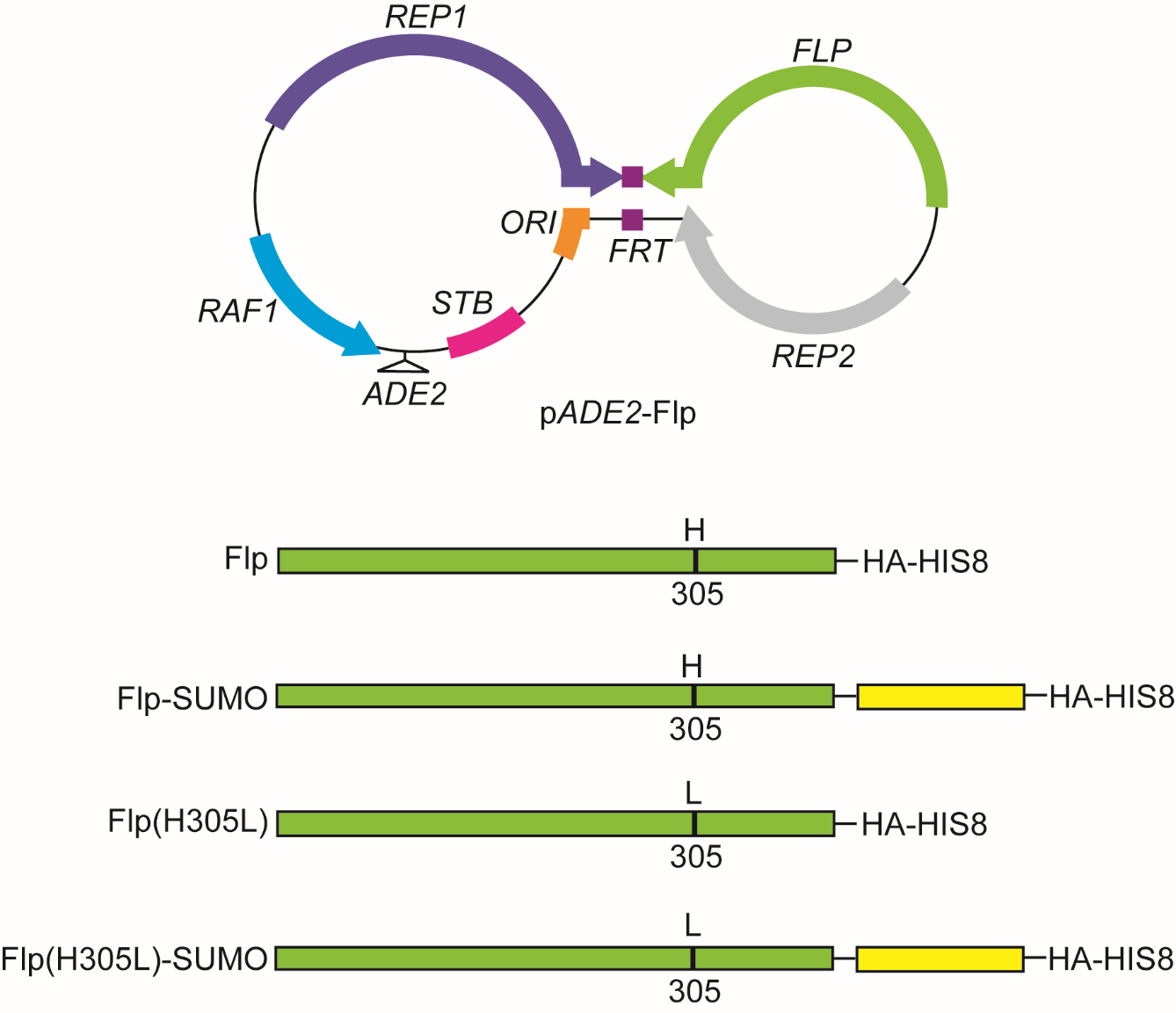
**

**S1 Fig. General characteristics of plasmids expressing Flp or modified forms of Flp are outlined.** The plasmid p*ADE2*-Flp, schematically diagrammed at the top, was a derivative of the 2-micron plasmid containing an insertion of the *ADE2* gene at the unique HpaI site within the plasmid genome [1,2]. In addition, the plasmid was engineered to express a version of Flp fused to an HA-His8 epitope at its carboxyl-terminus. Except for these modifications, the rest of the plasmid backbone was the same as that of the native 2-micron circle. The 599 bp inverted repeat sequences, within which the *FRT* sites are embedded, are shown by the horizontal parallel lines. The Flp-*FRT* recombination system and the Rep1-Rep2-*STB* partitioning system were functional in this multi-copy plasmid. In the parent form of p*ADE2*-Flp utilized for the present study, the 2-micron plasmid backbone was in the A-form [3]. Flp-mediated recombination in yeast between the head-to-head *FRT* sites of the plasmid could generate the B-form, and promote A 🡪 B and B 🡪 A interconversions (see S3 Fig). In p*ADE2*-Flp-SUMO, the *FLP* gene was modified to express a Flp-SUMO hybrid protein in which the mature form of SUMO (amino acids 1-96) was fused to the carboxyl-terminus of Flp via a short peptide linker: Ala-Ser-Gly_4_-Ser. The fusion protein harbored at its carboxyl-terminus the same HA-His8 tag as that fused to Flp. In the p*ADE2*-Flp(H305L) and p*ADE2*-Flp(H305L)-SUMO plasmid derivatives, the active site His-305 in Flp was replaced by leucine. The variants containing the H305L substitution were competent in strand cleavage but were strongly defective in strand joining. The Flp and SUMO moieties are color coded by green and yellow, respectively.

1. Ma CH, Cui H, Hajra S, Rowley PA, Fekete C, et al. (2013) Temporal sequence and cell cycle cues in the assembly of host factors at the yeast 2 micron plasmid partitioning locus. Nucleic Acids Res 41: 2340-2353.

2. Tsalik EL, Gartenberg MR (1998) Curing Saccharomyces cerevisiae of the 2 micron plasmid by targeted DNA damage. Yeast 14: 847-852.

3. Hartley JL, Donelson JE (1980) Nucleotide sequence of the yeast plasmid. Nature 286: 860-865.
