# Supplementary material for: A Flp-SUMO hybrid recombinase reveals multi-layered copy number control of a selfish DNA element through post-translational modification
Source: PLoS Genet. 2019 Jun 26;15(6):e1008193. doi: 10.1371/journal.pgen.1008193 (PMC6594588; doi:10.1371/journal.pgen.1008193)
Supplement: S2 Fig — The copy numbers of the native 2-micron plasmid in [Cir+] strains and of pADE2-Flp in [Cir0] strains (lacking the native plasmid) were estimated by real-time PCR essentially as described previously by Chen et al. [31]. In order that the present and published values are directly comparable, a 65 bp plasmid region and the Y’-subtelomeric element (as reference amplicon) were amplified from total yeast DNA preparations using the primer pairs described by Chen et al. [31]. The sequences of the amplification primers (P1, P2 for plasmid; P3, P4 for chromosome reference) are listed. The relative copy numbers are plotted with a value of 1.0 assigned to 2-micron plasmid in the [Cir+] strain. While siz1Δ siz2Δ raised the copy number of pADE2-Flp and the native 2-micron plasmid significantly (*, p < 0.05), the relative increase was lower for pADE2-Flp. (DOCX) [file pgen.1008193.s002.docx]

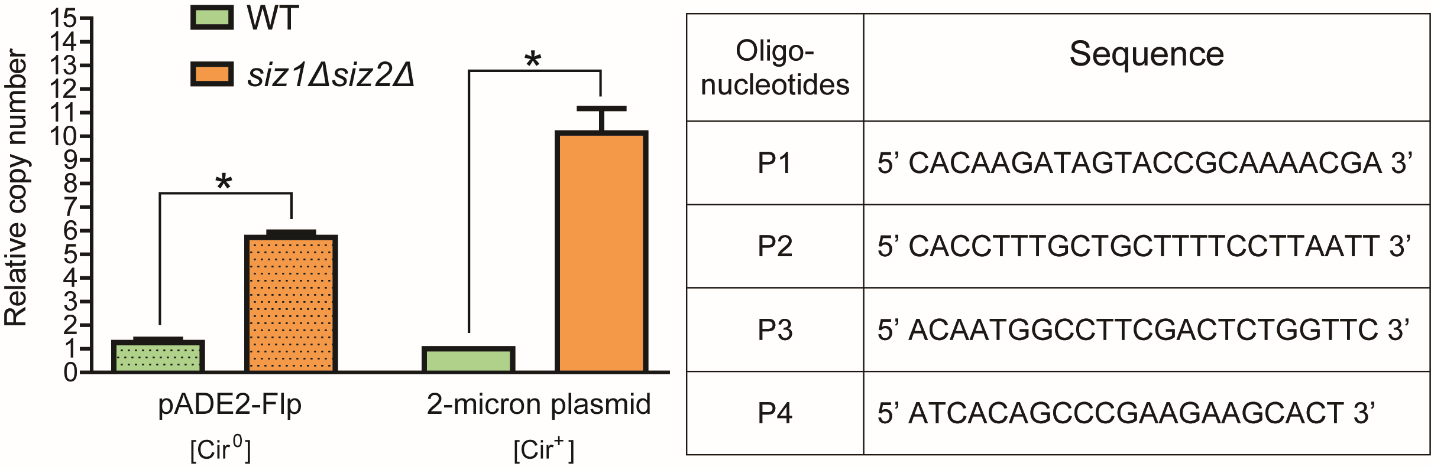


**S2 Fig. Plasmid copy numbers are compared in wild type and *siz1Δ* *siz2Δ* strains.** The copy numbers of the native 2-micron plasmid in [Cir^+^] strains and of p*ADE2*-Flp in [Cir^0^] strains (lacking the native plasmid) were estimated by real-time PCR essentially as described previously by Chen et al. [4]. In order that the present and published values are directly comparable, a 65 bp plasmid region and the Y’-subtelomeric element (as reference amplicon) were amplified from total yeast DNA preparations using the primer pairs described by Chen et al. [4]. The sequences of the amplification primers (P1, P2 for plasmid; P3, P4 for chromosome reference) are listed. The relative copy numbers are plotted with a value of 1.0 assigned to 2-micron plasmid in the [Cir^+^] strain. While *siz1Δ* *siz2Δ* raised the copy number of p*ADE2*-Flp and the native 2-micron plasmid significantly (*, p < 0.05), the relative increase was lower for p*ADE2*-Flp.

4. Chen XL, Reindle A, Johnson ES (2005) Misregulation of 2 micron circle copy number in a SUMO pathway mutant. Mol Cell Biol 25: 4311-4320.
