# Supplementary material for: A Flp-SUMO hybrid recombinase reveals multi-layered copy number control of a selfish DNA element through post-translational modification
Source: PLoS Genet. 2019 Jun 26;15(6):e1008193. doi: 10.1371/journal.pgen.1008193 (PMC6594588; doi:10.1371/journal.pgen.1008193)
Supplement: S3 Fig — A. The A-form of the pADE2-Flp-SUMO plasmid introduced into [Cir0] strains is shown schematically at the top (left). The B-form resulting from Flp-mediated recombination between the plasmid FRT sites is shown to its right. The two forms can be distinguished by the lengths of the PCR products formed with primer 1 (P1) plus primer 2 (P2) or primer 2’ (P2’) using isolated total yeast DNA as the template. Amplified DNA of the expected sizes formed by both primer pairs from pADE2-Flp-SUMO is consistent with the recombinase activity of Flp-SUMO expressed from it. The pADE2-Flp plasmid expressing wild type Flp served as a positive control for recombination. The larger product sizes from pADE2-Flp-SUMO are consistent with the increased length of the hybrid FLP-SUMO locus. B. Since pADE2-Flp-SUMO contains a 599 bp long inverted repeat sequence, each with an embedded FRT site, a strand break within one FRT may be repaired by using the second intact copy of the repeat as template. Resolution of a potential Holliday junction intermediate of repair in the crossover mode would produce the B-form plasmid from the parental A-form. In order to eliminate the possibility of repair-mediated A- to B-form conversion, a second recombination assay was performed in a [Cir0] strain containing a substrate plasmid in which two minimal FRT sites (each 34 bp long) bordered TRP1 in the head-to-tail orientation. The strain was engineered to express Flp-SUMO from a chromosomal locus under the control of the GAL promoter. Aliquots from overnight cultures grown under non-inducing (glucose) and inducing (galactose) conditions were plated on medium lacking uracil, and 3-day old colonies were replica-plated and grown on medium without tryptophan. The loss of the TRP1 marker in galactose-grown cells verifies FRT x FRT recombination mediated by Flp-SUMO. Expression of Flp-SUMO(Y343F) did not yield TRP1 deletion. (DOCX) [file pgen.1008193.s003.docx]

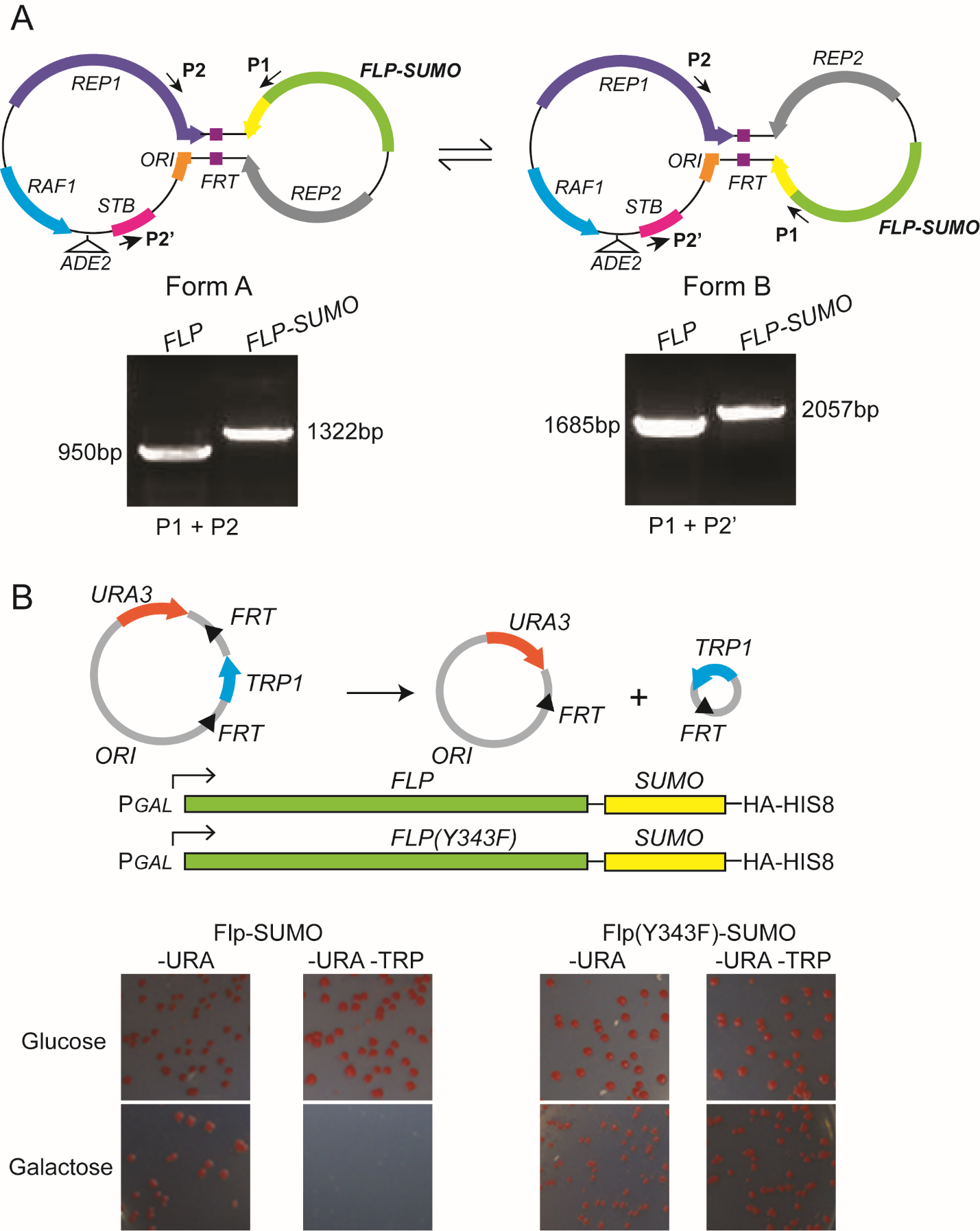
 **S3 Fig. Flp-SUMO is active in mediating recombination between *FRT* sites.** **A**. The A-form

of the p*ADE2*-Flp-SUMO plasmid introduced into [Cir^0^] strains is shown schematically at the top (left). The B-form resulting from Flp-mediated recombination between the plasmid *FRT* sites is shown to its right. The two forms can be distinguished by the lengths of the PCR products formed with primer 1 (P1) plus primer 2 (P2) or primer 2’ (P2’) using isolated total yeast DNA as the template. Amplified DNA of the expected sizes formed by both primer pairs from p*ADE2*-Flp-SUMO is consistent with the recombinase activity of Flp-SUMO expressed from it. The p*ADE2*-Flp plasmid expressing wild type Flp served as a positive control for recombination. The larger product sizes from p*ADE2*-Flp-SUMO are consistent with the increased length of the hybrid *FLP*-SUMO locus. **B**. Since p*ADE2*-Flp-SUMO contains a 599 bp long inverted repeat sequence, each with an embedded *FRT* site, a strand break within one *FRT* may be repaired by using the second intact copy of the repeat as template. Resolution of a potential Holliday junction intermediate of repair in the crossover mode would produce the B-form plasmid from the parental A-form. In order to eliminate the possibility of repair-mediated A- to B-form conversion, a second recombination assay was performed in a [Cir^0^] strain containing a substrate plasmid in which two minimal *FRT* sites (each 34 bp long) bordered *TRP1* in the head-to-tail orientation. The strain was engineered to express Flp-SUMO from a chromosomal locus under the control of the *GAL* promoter. Aliquots from overnight cultures grown under non-inducing (glucose) and inducing (galactose) conditions were plated on medium lacking uracil, and 3-day old colonies were replica-plated and grown on medium without tryptophan. The loss of the *TRP1* marker in galactose-grown cells verifies *FRT* x *FRT* recombination mediated by Flp-SUMO. Expression of Flp-SUMO(Y343F) did not yield *TRP1* deletion.
