# Supplementary material for: A Flp-SUMO hybrid recombinase reveals multi-layered copy number control of a selfish DNA element through post-translational modification
Source: PLoS Genet. 2019 Jun 26;15(6):e1008193. doi: 10.1371/journal.pgen.1008193 (PMC6594588; doi:10.1371/journal.pgen.1008193)
Supplement: S4 Fig — Native Flp and Flp-HA-His8 were purified to near homogeneity using E. coli expression systems [43–45]. The excision reaction (schematically illustrated at the top) was performed as outlined in the legend to (Fig 6A and 6B). The EcoRV plus NdeI digestion products formed from the substrate plasmid and the excision circles are indicated by ‘S’ and ‘P’, respectively. (DOCX) [file pgen.1008193.s004.docx]

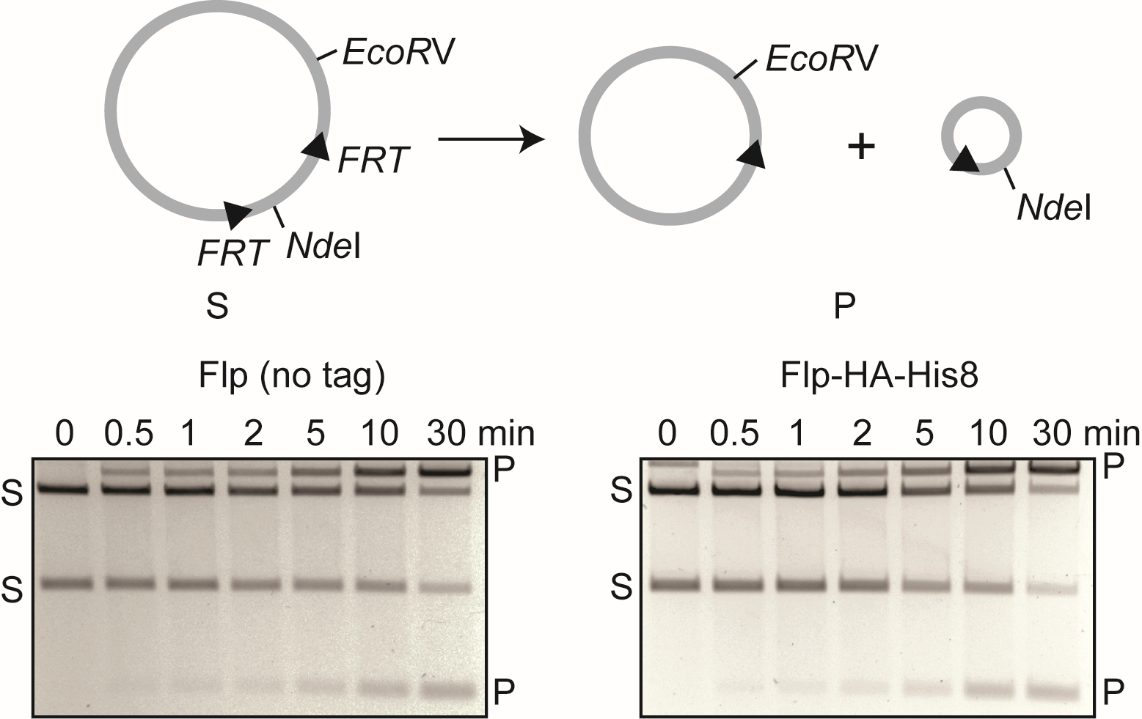


**S4 Fig. Native Flp and Flp epitope-tagged at its carboxyl-terminus are equally active in recombination *in vitro*.** Native Flp and Flp-HA-His8 were purified to near homogeneity using *E. coli* expression systems [5-7]. The excision reaction (schematically illustrated at the top) was performed as outlined in the legend to Fig 6A-C. The EcoRV plus NdeI digestion products formed from the substrate plasmid and the excision circles are indicated by ‘S’ and ‘P’, respectively.

5. Lee J, Whang I, Jayaram M (1996) Assembly and orientation of Flp recombinase active sites on two-, three- and four-armed DNA substrates: implications for a recombination mechanism. J Mol Biol 257: 532-549.

6. Meyer-Leon L, Gates CA, Attwood JM, Wood EA, Cox MM (1987) Purification of the FLP site-specific recombinase by affinity chromatography and re-examination of basic properties of the system. Nucleic Acids Res 15: 6469-6488.

7. Parsons RL, Evans BR, Zheng L, Jayaram M (1990) Functional analysis of Arg-308 mutants of Flp recombinase. Possible role of Arg-308 in coupling substrate binding to catalysis. J Biol Chem 265: 4527-4533.
