# Supplementary material for: A Flp-SUMO hybrid recombinase reveals multi-layered copy number control of a selfish DNA element through post-translational modification
Source: PLoS Genet. 2019 Jun 26;15(6):e1008193. doi: 10.1371/journal.pgen.1008193 (PMC6594588; doi:10.1371/journal.pgen.1008193)
Supplement: S1 Table — The genotypes of the yeast strains used in the present study along with the figures/table depicting the experimental results obtained with them are listed. (DOCX) [file pgen.1008193.s005.docx]

**S1 Table. Yeast strains and their relevant features.** The genotypes of the yeast strains used in the present study along with the figures/table depicting the experimental results obtained with them are listed.

| **Strains** | **Genotype or salient features** | **Relevant**  **Figures**  **/Tables** |
| --- | --- | --- |
| MJY5101 | *MAT****a*** *ade2-1::*p*ADE2-*Flp *his3-11 leu2-3,112 trp1 ura3-1* [Cir^0^] | Figure 2;  Table S3 |
| MJY5102 | *MAT****a*** *ade2-1::*p*ADE2-*Flp *his3-11::siz2Δ::HIS3 leu2-3,112 ::siz1Δ::LEU2 trp1 ura3-1* [Cir^0^] | Figure 2;  Table S3 |
| MJY5103 | *MAT****a*** *ade2-1::*p*ADE2-*Flp-SUMO *his3-11 leu2-3,112 trp1 ura3-1* [Cir^0^] | Figure 2;  Table S3 |
| MJY5104 | *MAT****a*** *ade2-1::*p*ADE2-*Flp-SUMO *his3-11::siz2Δ::HIS3 leu2-3,112 ::siz1Δ::LEU2 trp1 ura3-1* [Cir^0^] | Figure 2;  Table S3 |
| MJY5105 | *MAT****a*** *ade2-1::*p*ADE2-*Flp(H305L) *his3-11 leu2-3,112 trp1 ura3-1* [Cir^0^] | Figure 2;  Table S3 |
| MJY5106 | *MAT****a*** *ade2-1::*p*ADE2-*Flp(H305L) *his3-11::siz2Δ::HIS3 leu2-3,112 ::siz1Δ::LEU2 trp1 ura3-1* [Cir^0^] | Figure 2;  Table S3 |
| MJY5107 | *MAT****a*** *ade2-1 his3-11 leu2-3,112:: P_GAL_*-Flp(H305L)*::LEU2 trp1 ura3-1 FRT::KanMX6* [Cir^0^] | Figure 3 |
| MJY5108 | *MAT****a*** *ade2-1 his3-11 leu2-3,112:: P_GAL_*-Flp(H305L)*::LEU2 trp1::mus81Δ::TRP1 ura3-1 FRT::KanMX6* [Cir^0^] | Figure 3 |
| MJY5109 | *MAT****a*** *ade2-1 his3-11 leu2-3,112:: P_GAL_*-Flp(H305L)*::LEU2 trp1 ura3-1 yen1Δ::NatMX FRT::KanMX6* [Cir^0^] | Figure 3 |
| MJY5110 | *MAT****a*** *ade2-1 his3-11 leu2-3,112:: P_GAL_*-Flp(H305L)*::LEU2 trp1::mus81Δ::TRP1 ura3-1 yen1Δ::NatMX FRT::KanMX6* [Cir^0^] | Figure 3 |
| MJY5111 | *MAT****a*** *ade2-1 his3-11 leu2-3,112:: P_GAL_*-Flp(H305L)*::LEU2 trp1 ura3-1 FRT::KanMX6* [Cir^+^] | Figure 3 |
| MJY5112 | *MAT****a*** *ade2-1 his3-11 leu2-3,112:: P_GAL_*-Flp(H305L)*::LEU2 trp1::mus81Δ::TRP1 ura3-1 FRT::KanMX6* [Cir^+^] | Figure 3 |
| MJY5113 | *MAT****a*** *ade2-1 his3-11 leu2-3,112:: P_GAL_*-Flp(H305L)*::LEU2 trp1 ura3-1 yen1Δ::NatMX FRT::KanMX6* [Cir^+^] | Figure 3 |
| MJY5114 | *MAT****a*** *ade2-1 his3-11 leu2-3,112:: P_GAL_*-Flp(H305L)*::LEU2 trp1::mus81Δ::TRP1 ura3-1 yen1Δ::NatMX FRT::KanMX6* [Cir^+^] | Figure 3 |
| MJY5115 | *MAT****a*** *ade2-1 his3-11 leu2-3,112:: P_GAL_*-Flp(H305L)-SUMO*::LEU2 trp1 ura3-1 FRT::KanMX6* [Cir^+^] | Figure 3 |
| MJY5116 | *MAT****a*** *ade2-1 his3-11 leu2-3,112:: P_GAL_*-Flp(H305L)-SUMO*::LEU2 trp1::mus81Δ::TRP1 ura3-1 FRT::KanMX6* [Cir^+^] | Figure 3 |
| MJY5117 | *MAT****a*** *ade2-1 his3-11 leu2-3,112:: P_GAL_*-Flp(H305L)-SUMO*::LEU2 trp1 ura3-1 yen1Δ::NatMX FRT::KanMX6* [Cir^+^] | Figure 3 |
| MJY5118 | *MAT****a*** *ade2-1 his3-11 leu2-3,112:: P_GAL_*-Flp(H305L)-SUMO*::LEU2 trp1::mus81Δ::TRP1 ura3-1 yen1Δ::NatMX FRT::KanMX6* [Cir^+^] | Figure 3 |
| MJY5119 | *MAT****a*** *ade2-1::*p*ADE2-*Flp *his3-11::slx5Δ::HIS3 leu2-3,112 trp1 ura3-1* [Cir^0^] | Figure 4;  Table S3 |
| MJY5120 | *MAT****a*** *ade2-1::*p*ADE2-*Flp *his3-11 leu2-3,112 trp1 ura3-1::slx8Δ::URA3* [Cir^0^] | Figure 4;  Table S3 |
| MJY5121 | *MAT****a*** *ade2-1::*p*ADE2-*Flp-SUMO *his3-11::slx5Δ::HIS3 leu2-3,112 trp1 ura3-1* [Cir^0^] | Figure 4;  Table S3 |
| MJY5122 | *MAT****a*** *ade2-1::*p*ADE2-*Flp-SUMO *his3-11 leu2-3,112 trp1 ura3-1::slx8Δ::URA3* [Cir^0^] | Figure 4;  Table S3 |
| MJY5123 | *MAT****a*** *ade2-1 his3-11 leu2-3,112 trp1:: P_GAL_*-Flp-SUMO-HA-His8 *ura3-1* [Cir^0^] | Figure 5 |
| MJY5124 | *MAT****a*** *ade2-1 his3-11::siz2Δ::HIS3 leu2-3,112::siz1Δ::LEU2 trp1:: P_GAL_*-Flp-SUMO-HA-His8 *ura3-1* [Cir^0^] | Figure 5 |
| MJY5125 | *MAT****a*** *ade2-1 his3-11::slx5Δ::HIS3 leu2-3,112 trp1:: P_GAL_*-Flp-SUMO-HA-His8 *ura3-1* [Cir^0^] | Figure 5 |
| MJY5126 | *MAT****a*** *ade2-1::his3-11 leu2-3,112 trp1:: P_GAL_*-Flp-SUMO-HA-His8 *ura3-1::slx8Δ::URA3* [Cir^0^] | Figure 5 |
| MJY5127 | *MAT****a*** *ade2-1 his3-11 leu2-3,112 trp1:: P_GAL_*-Flp-HA-His8 *ura3-1* [Cir^0^] :: pCM422 | Figure 9 |
| MJY5128 | *MAT****a*** *ade2-1 his3-11::siz2Δ::HIS3 leu2-3,112::siz1Δ::LEU2 trp1:: P_GAL_*-Flp-HA-His8 *ura3-1* [Cir^0^] :: pCM422 | Figure 9 |
| MJY5129 | *MAT****a*** *ade2-1 his3-11 leu2-3,112 trp1:: P_GAL_*-Flp-SUMO-HA-His8 *ura3-1* [Cir^0^] :: pCM422 | Figure 9 |
| MJY5130 | *MAT****a*** *ade2-1 his3-11::siz2Δ::HIS3 leu2-3,112::siz1Δ::LEU2 trp1:: P_GAL_*-Flp-SUMO-HA-His8 *ura3-1* [Cir^0^] :: pCM422 | Figure 9 |
| MJY5131 | *MAT****a*** *ade2-1 his3-11 leu2-3,112 trp1 ura3-1* [Cir^0^] :: pCM422 | Figure 9 |
| MJY5132 | *MAT****a*** *ade2-1 his3-11::siz2Δ::HIS3 leu2-3,112::siz1Δ::LEU2 trp1:: ura3-1* [Cir^0^] :: pCM422 | Figure 9 |
