# Supplementary material for: A Flp-SUMO hybrid recombinase reveals multi-layered copy number control of a selfish DNA element through post-translational modification
Source: PLoS Genet. 2019 Jun 26;15(6):e1008193. doi: 10.1371/journal.pgen.1008193 (PMC6594588; doi:10.1371/journal.pgen.1008193)
Supplement: S2 Table — Yeast and bacterial plasmids utilized in this work and their relevant features are summarized. The figures/table containing experimental data obtained using these plasmids are indicated. The Flp and Flp-SUMO proteins and their H305L variants expressed from the yeast plasmids contained the HA-His8 tag at their carboxyl-terminus. The pCM66 and pCM331 integrative plasmids were inserted into the leu2 locus of the experimental yeast strains. The pBAD33-derived plasmids were used for the expression and purification of Flp with or without the carboxyl-terminal HA-His8 tag as well as Flp-SUMO containing this tag. The pBAD24-derived plasmid served as the substrate for in vitro recombination assays. (DOCX) [file pgen.1008193.s006.docx]

**S2 Table. Plasmids.** Yeast and bacterial plasmids utilized in this work and their relevant features are summarized. The figures/table containing experimental data obtained using these plasmids are indicated. The Flp and Flp-SUMO proteins and their H305L variants expressed from the yeast plasmids contained the HA-His8 tag at their carboxyl-terminus. The pCM66 and pCM331 integrative plasmids were inserted into the *leu2* locus of the experimental yeast strains. The pBAD33-derived plasmids were used for the expression and purification of Flp with or without the carboxyl-terminal HA-His8 tag as well as Flp-SUMO containing this tag. The pBAD24-derived plasmid served as the substrate for *in vitro* recombination assays.

| **Plasmids** | | **Relevant**  **Figures**  **/Tables** |
| --- | --- | --- |
| p*ADE2*-Flp | 2-micron-derived plasmid containing *ADE2* at the *Hpa*I site | Figures 2, 4 and S2  Table S3 |
| p*ADE2*-Flp-SUMO | p*ADE2*-Flp derivative with SUMO (amino acids1-96) fused to the carboxyl-terminus of Flp | Figures 2 and 4  Table S3 |
| p*ADE2*-Flp(H305L) | p*ADE2*-Flp containing H305L substitution within Flp | Figure 2  Table S3 |
| pCM66 | *P_GAL1_-*Flp(H305L)-*LEU2* cloned in pUC19 | Figure 3 |
| pCM331 | *P_GAL1_-*Flp(H305L)-SUMO-*LEU2* cloned in pUC19 | Figure 3 |
| pBAD24-*FRT-FRT* | Two *FRT* sites in head-to-tail orientation cloned into pBAD24 | Figures 6 and S4 |
| pBAD33-Flp | Plasmid for arabinose-inducible expression of native Flp | Figures 6 and 7 |
| pCM327 | Plasmid for arabinose-inducible expression of Flp-HA-His8 | Figures 6 and 7 |
| pCM318 | Plasmid for arabinose-inducible expression of Flp-SUMO-HA-His8 | Figures 6 and 7 |
| pCM374 | Plasmid for arabinose-inducible expression of Flp(R191A)-HA-His8 | Figures 8 |
| pCM375 | Plasmid for arabinose-inducible expression of Flp(Y343F)-HA-His8 | Figures 8 |
| pCM376 | Plasmid for arabinose-inducible expression of Flp(R191A)-SUMO-HA-His8 | Figures 8 |
| pCM377 | Plasmid for arabinose-inducible expression of Flp(Y343F)-SUMO-HA-His8 | Figures 8 |
| pCM422 | pRS414 derivative (*RAD52*-*YFP* *TRP1*) | Figure 9 |
| pCM189 | p*GAL1*-Flp-SUMO-HA-His8 (*CEN*, *LEU2*) | Figure S3 |
| pCM204 | p*GAL1*-Flp(Y343F)-SUMO-HA-His8 (*CEN*, *LEU2*) | Figure S3 |
| pCM232 | pRS416-*FRT-TRP1-FRT-URA3* (*FRT* sites in head-to-tail orientation) | Figure S3 |
