# Supplementary material for: A Flp-SUMO hybrid recombinase reveals multi-layered copy number control of a selfish DNA element through post-translational modification
Source: PLoS Genet. 2019 Jun 26;15(6):e1008193. doi: 10.1371/journal.pgen.1008193 (PMC6594588; doi:10.1371/journal.pgen.1008193)

**S3 Table. Nibbled colonies and mini-colonies induced by p*ADE2*-Flp or its derivatives are quantified in wild type and mutant strains.** Representative colony types observed are shown at the top: **A**. smooth white (normal) colonies harboring an *ADE2* containing reporter plasmid; **B**. plasmid containing nibbled and growth-stunted (mini) colonies; **C**. a smooth red colony formed by a plasmid-free cell (left) and a colony with a smooth red (plasmid-free) sector and a nibbled white (plasmid containing) sector (right). The colony morphologies of the wild type [Cir^0^], *siz1Δ* *siz2Δ* [Cir^0^], *slx5Δ* [Cir^0^] and *slx8Δ* [Cir^0^] strains harboring the indicated plasmids were quantitated from the assays depicted in Figs 2, 4 and similar assays. The listed values represent the combined averages from cell populations plated on YEPD medium at n = 0 (overnight culture in selective medium lacking adenine) and at n = 10 (after n = 0 cells were grown non-selectively in liquid medium for 10 generations). The liquid cultures were incubated at 30°C. The plate cultures derived from n = 0 and n = 10 liquid cultures were grown at 26°C (5 days) before screening colony morphology (a total of > 800 colonies from n = 0 and n = 10 plates for each assay). As explained in the legend to Fig 2, only white colonies and the white patches within sectored colonies were scored for the presence of nibbled edges. Mini-colonies were so designated when they were conspicuously smaller than the average sized colonies on 5-day incubated plates. Completely red colonies, indicating plasmid loss before plating, were omitted from consideration. WT = wild type strain.


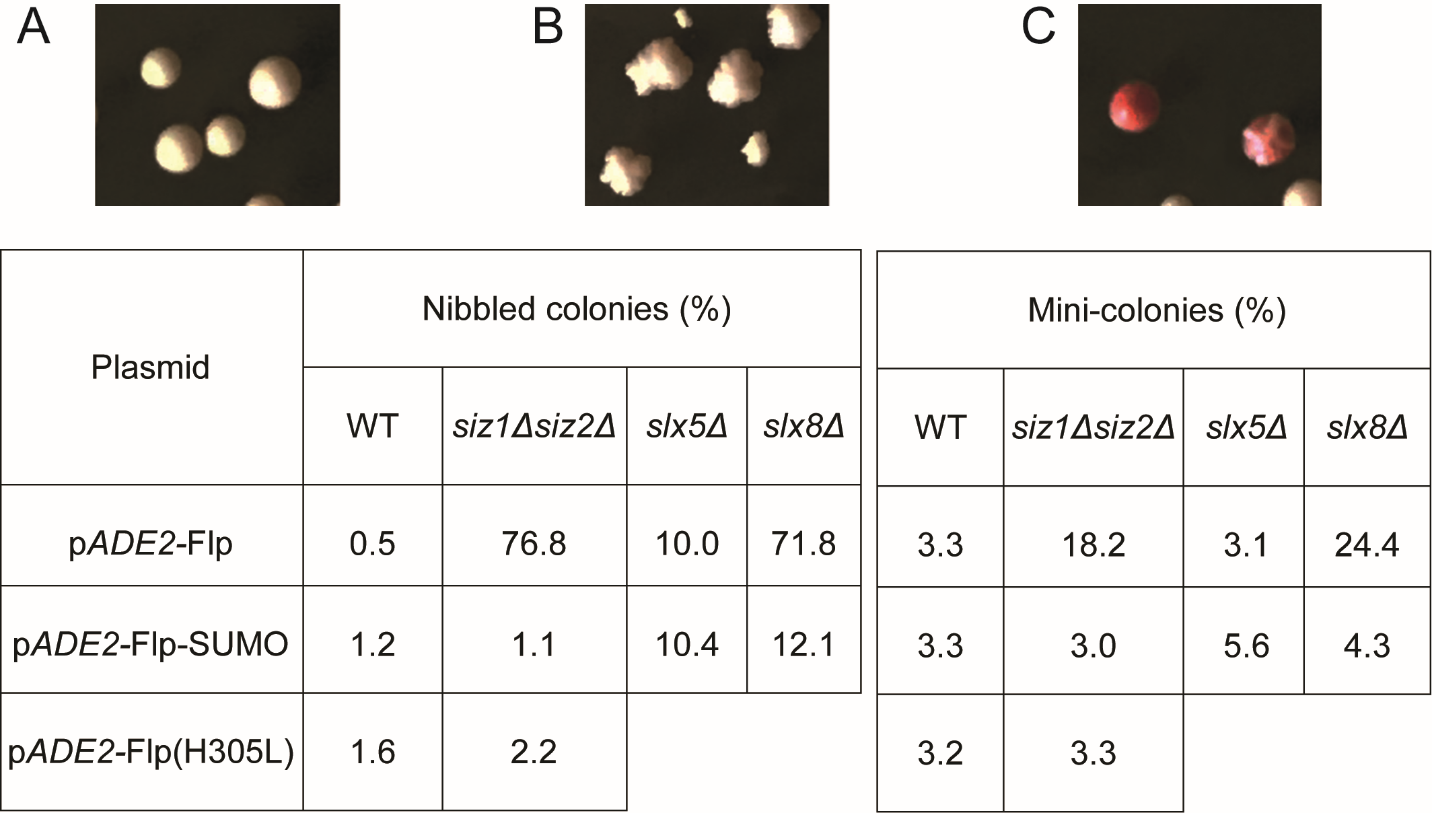

Supplement: S3 Table — Representative colony types observed are shown at the top: A. smooth white (normal) colonies harboring an ADE2 containing reporter plasmid; B. plasmid containing nibbled and growth-stunted (mini) colonies; C. a smooth red colony formed by a plasmid-free cell (left) and a colony with a smooth red (plasmid-free) sector and a nibbled white (plasmid containing) sector (right). The colony morphologies of the wild type [Cir0], siz1Δ siz2Δ [Cir0], slx5Δ [Cir0] and slx8Δ [Cir0] strains harboring the indicated plasmids were quantitated from the assays depicted in Figs 2 and 4 and similar assays. The listed values represent the combined averages from cell populations plated on YEPD medium at n = 0 (overnight culture in selective medium lacking adenine) and at n = 10 (after n = 0 cells were grown non-selectively in liquid medium for 10 generations). The liquid cultures were incubated at 30°C. The plate cultures derived from n = 0 and n = 10 liquid cultures were grown at 26°C (5 days) before screening colony morphology (a total of > 800 colonies from n = 0 and n = 10 plates for each assay). As explained in the legend to Fig 2, only white colonies and the white patches within sectored colonies were scored for the presence of nibbled edges. Mini-colonies were so designated when they were conspicuously smaller than the average sized colonies on 5-day incubated plates. Completely red colonies, indicating plasmid loss before plating, were omitted from consideration. WT = wild type strain. (DOCX) [file pgen.1008193.s007.docx]
