# Supplementary material for: A Flp-SUMO hybrid recombinase reveals multi-layered copy number control of a selfish DNA element through post-translational modification
Source: PLoS Genet. 2019 Jun 26;15(6):e1008193. doi: 10.1371/journal.pgen.1008193 (PMC6594588; doi:10.1371/journal.pgen.1008193)
Supplement: S4 Table — The distances between α-carbon atoms assembled in panel A are for a Flp monomer (bound adjacent to a cleaved scissile phosphate). In this structure (PDB, 1M6X), the polypeptide chain ends with Arg-422; Ile-423 is not visible [70]. Similar distances arranged in panel B are for a Flp monomer (bound adjacent to an uncleaved scissile phosphate). The carboxyl-terminal I-423 is visible in this structure (PDB, 1FLO) [59]. (DOCX) [file pgen.1008193.s008.docx]

**S4 Table. Relative dispositions of Lys-375 and the carboxyl-terminus of Flp with respect to the catalytic hexad residues of the active site are displayed.** The distances between α-carbon atoms assembled in panel **A** are for a Flp monomer (bound adjacent to a cleaved scissile phosphate). In this structure (PDB, 1M6X), the polypeptide chain ends with Arg-422; Ile-423 is not visible [8]. Similar distances arranged in panel **B** are for a Flp monomer (bound adjacent to an uncleaved scissile phosphate). The carboxyl-terminal I-423 is visible in this structure (PDB, 1FLO) [9].

8. Conway AB, Chen Y, Rice PA (2003) Structural plasticity of the Flp-Holliday junction complex. J Mol Biol 326: 425-434.


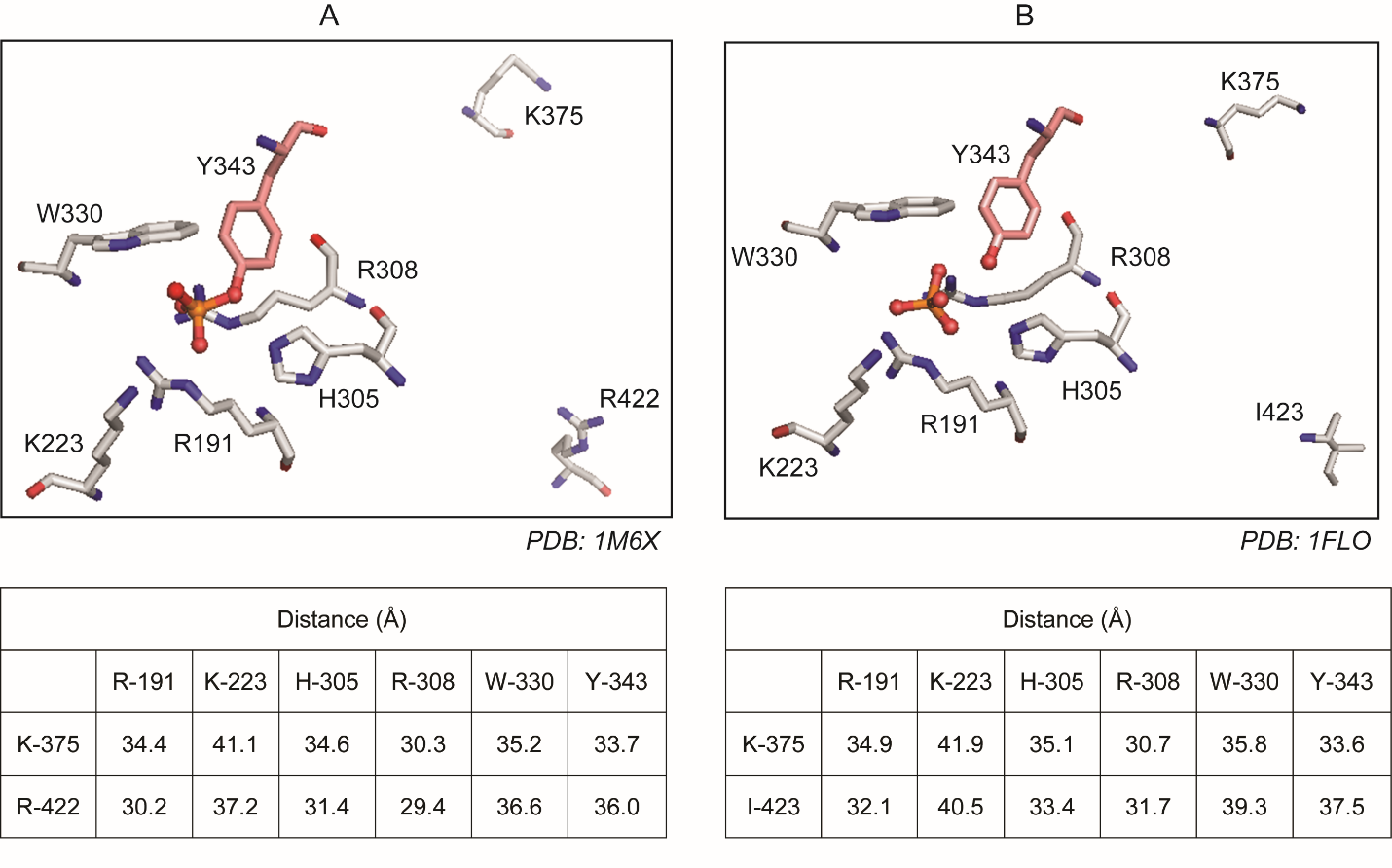
9. Chen Y, Narendra U, Iype LE, Cox MM, Rice PA (2000) Crystal structure of a Flp recombinase-Holliday junction complex: assembly of an active oligomer by helix swapping. Mol Cell 6: 885-897.
